# Metagenomic identification, purification and characterisation of the *Bifidobacterium adolescentis* BgaC β-galactosidase

**DOI:** 10.1007/s00253-020-11084-y

**Published:** 2021-01-11

**Authors:** Daniel Mehabie Mulualem, Christy Agbavwe, Lesley A. Ogilvie, Brian V. Jones, Michelle Kilcoyne, Conor O’Byrne, Aoife Boyd

**Affiliations:** 1 Discipline of Microbiology, School of Natural Sciences, National University of Ireland Galway, Galway, Ireland; 2 Max Planck Institute for Molecular Genetics, Berlin, Germany; 3 Department of Biology & Biochemistry, University of Bath, Bath, UK

**Keywords:** Enzyme screening, Metagenomic library, β-Galactosidase, *Bifidobacterium*, Transglycosylation

## Abstract

Members of the human gut microbiota use glycoside hydrolase (GH) enzymes, such as β-galactosidases, to forage on host mucin glycans and dietary fibres. A human faecal metagenomic fosmid library was constructed and functionally screened to identify novel β-galactosidases. Out of the 16,000 clones screened, 30 β-galactosidase-positive clones were identified. The β-galactosidase gene found in the majority of the clones was *BAD_1582* from *Bifidobacterium adolescentis*, subsequently named *bgaC*. This gene was cloned with a hexahistidine tag, expressed in *Escherichia coli* and His-tagged-BgaC was purified using Ni^2+^-NTA affinity chromatography and size filtration. The enzyme had optimal activity at pH 7.0 and 37 °C, with a wide range of pH (4–10) and temperature (0–40 °C) stability. It required a divalent metal ion co-factor; maximum activity was detected with Mg^2+^, while Cu^2+^ and Mn^2+^ were inhibitory. Kinetic parameters were determined using ortho-nitrophenyl-β-d-galactopyranoside (ONPG) and lactose substrates. BgaC had a *V*_max_ of 107 μmol/min/mg and a *K*_*m*_ of 2.5 mM for ONPG and a *V*_max_ of 22 μmol/min/mg and a *K*_*m*_ of 3.7 mM for lactose. It exhibited low product inhibition by galactose with a *K*_*i*_ of 116 mM and high tolerance for glucose (66% activity retained in presence of 700 mM glucose). In addition, BgaC possessed transglycosylation activity to produce galactooligosaccharides (GOS) from lactose, as determined by TLC and HPLC analysis. The enzymatic characteristics of *B. adolescentis* BgaC make it an ideal candidate for dairy industry applications and prebiotic manufacture.

**Key points**

• *Bifidobacterium adolescentis BgaC β-galactosidase was selected from human faecal metagenome.*

• *BgaC possesses sought-after properties for biotechnology, e.g. low product inhibition.*

• *BgaC has transglycosylation activity producing prebiotic oligosaccharides.*

**Supplementary Information:**

The online version contains supplementary material available at 10.1007/s00253-020-11084-y.

**Figure:**
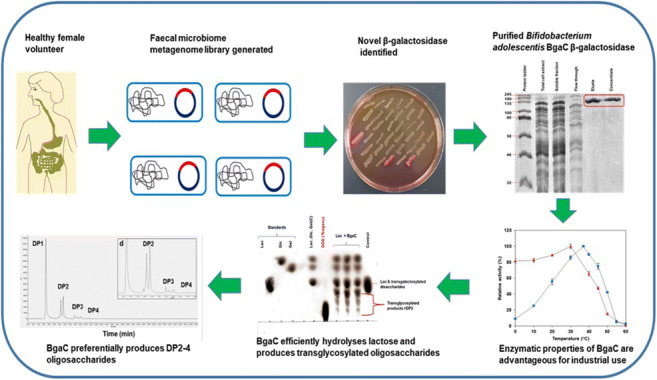
Graphical Abstract

## Introduction

β-Galactosidases (E.C.3.2.1.23) are enzymes that catalyse the hydrolytic cleavage of galactose residues from the non-reducing end of β-galactosides. β-Galactosidases belong to the six glycoside hydrolase families of GH 1, GH 2, GH 35, GH 42, GH 59, and GH 147 within the Carbohydrate-Active enZymes (CAZy) database (http://www.cazy.org/) (Lombard et al. 2014). In the past decades, there has been a growing interest in identification and characterisation of β-galactosidases, primarily for application in dairy industries due to their hydrolysis of lactose into glucose and galactose (Adam et al. 2004). Lactose hydrolysis reduces crystallisation problems resulting from the low solubility of lactose and increases the sweetness of dairy products (Gänzle et al. 2008), and the reduced lactose content in whey by-product decreases water pollution (Mawson 1994). Furthermore, the demand for lactose-free dairy products has increased due to the growing number of lactose-intolerant people worldwide, reported to be between 2 and 70% in Europe and the USA and approximately 100% in Asia (Vandenplas 2015). In addition, some β-galactosidases can carry out transglycosylation, whereby they transfer galactose residues to lactose acceptors to synthesise galactooligosaccharides (GOS) with various linkages and degrees of polymerisation (Reuter et al. 1999; Torres et al. 2010). Several in vitro and in vivo studies have shown that GOS selectively stimulates the growth of beneficial *Bifidobacteria* and *Lactobacillus* species in the gut (Torres et al. 2010; Walton et al. 2012), which is a valuable characteristic for infant milk formulas. Furthermore, GOS administered together with bacteria such as *Bifidobacterium* (synbiotics) (Gibson and Roberfroid 1995) can promote proliferation of these exogenously supplemented probiotics (Kolida and Gibson 2011). Thus, the biotechnological applications of β-galactosidases encompass production of lactose-free dairy products and production of GOS with prebiotic properties.

Commercially used β-galactosidases are most commonly obtained from *Kluveromyces* spp. and *Aspergillus* spp. However, these β-galactosidases have some undesirable properties, such as a low affinity for lactose and product inhibition by galactose at low *K*_*i*_ (Erich et al. 2015). There is a need for the discovery of novel β-galactosidases which do not have these characteristics. In addition, there is demand for novel β-galactosidases with wider ranges of thermal and pH stability to suit industrial downstream processes and that can synthesise GOS with different types of linkages and degrees of polymerisation.

Metagenomics has uncovered genomes of uncultivated microorganisms from untapped environments leading to the discovery of natural products and enzymes with unique properties (Simon and Daniel 2011). Through function-based and sequence-based screening of metagenomes from diverse habitats, such as marine sediments, compost, desert sand and hydrothermal vents, valuable enzymes for biotechnology, food and pharmaceutical industries have been identified, including lipases, proteases and glycoside hydrolases (Lee et al. 2004; Neveu et al. 2011; Uchiyama et al. 2013). Human gut microbiome metagenomic libraries have revealed novel glycoside hydrolases (Cecchini et al. 2013; Tasse et al. 2010) and β-galactosidases with unique thermo-tolerant and alkaline-tolerant properties have been identified in environmental metagenomic libraries (Cheng et al. 2017; Liu et al. 2019; Wierzbicka-Woś et al. 2013; Zhang et al. 2013).

In this study, a human faecal metagenomic fosmid library was used to identify β-galactosidases by functional screening. Clones with β-galactosidase activity were sequenced and a β-galactosidase enzyme of *Bifidobacterium adolescentis*, BAD_1582, was identified, which we have named BgaC. The enzyme was recombinantly expressed with a hexahistidine tag, purified, and the properties of the expressed enzyme were characterised. BgaC exhibited efficient hydrolysis of lactose and transglycosylation activity to produce oligosaccharides at low concentrations of lactose. These properties make the enzyme an ideal candidate for large-scale enzymatic hydrolysis of lactose and synthesis of potential prebiotic oligosaccharides in dairy and pharmaceutical industries.

## Material and methods

All chemicals were analytical grade and purchased from Sigma-Aldrich/Merck, unless otherwise stated.

### Bacterial strains, plasmids, fosmids and growth conditions

The *Escherichia coli* strains, fosmids and plasmids used in this study are shown in the Electronic Supplementary Material, Table S1. *E. coli* strains were grown at 37 °C in LB agar and broth with aeration. Antibiotics were added at the following final concentrations: chloramphenicol (12.5 μg/ml) and ampicillin (120 μg/ml).

### Construction of human faecal metagenome library

A human faecal microbiome metagenome library was constructed using the Copy Control Fosmid Library Production Kit with pCC1FOS Vector (Epicentre) according to the manufacturer’s instructions (Agbavwe 2017). This kit utilises a strategy of cloning randomly sheared, end-repaired DNA. A healthy, 27-year-old, female volunteer, consuming a Western diet (omnivorous), provided a fresh faecal sample for this study. The volunteer did not take any antibiotics or other drugs known to influence the faecal microbiota within the 6-month period prior to the study. The stool sample was homogenised and large particles removed by centrifugation. Bacterial cells were physically separated from host cells by density gradient layering (Nycodenz Axis Shield 1002424) (Ford and Rickwood 1982). DNA from the bacterial cells was then isolated using conventional DNA extraction methods.

Representative community DNA of the faecal microbiome was purified, and sheared, and the fragments were end-repaired to produce 5′-phosphorylated blunt ends. The desired size range (approximately 40 kb) of end-repaired DNA was isolated using agarose gel electrophoresis and ligated to the pCC1FOS vector. The ligated DNA was packaged into lambda phage and introduced into *E. coli* EPI300-T1^R^. The infected *E. coli* EPI300-T1^R^ cells were plated on selective LB agar media and allowed to grow overnight. The clones which harboured the ligated fosmids were picked, induced to high copy number using l-arabinose auto induction solution, pooled to generate the human faecal microbiome metagenome library and stored in aliquots at 15% glycerol at − 80 °C. These aliquots were used for targeted gene identification via function-based screening.

### Functional screening of putative β-galactosidase-encoding clones

The human faecal microbiome metagenome library was screened for pink colonies expressing a functional β-galactosidase on MacConkey lactose agar. A vial of stock human faecal microbiome metagenome library was thawed on ice, and a serial dilution which provided approximately 500 colonies per plate was prepared in LB broth. One hundred microliters was plated on MacConkey agar supplemented with lactose, Cm and arabinose. β-Galactosidase-positive clones were identified based on their pink morphology while β-galactosidase-negative clones were colourless. Clones that appeared pink on the MacConkey agar were picked and streaked on LB agar containing Cm, arabinose and the chromogenic substrate 5-bromo-4-chloro-3-indolyl-β-galactopyranoside (X-Gal) at final concentration of 40 μg ml^−1^ for confirmatory test, and positive clones were identified by formation of blue colonies. The Miller assay (Miller 1972) with ONPG substrate was conducted on positive clones to determine the β-galactosidase activity of the positive fosmid clones.

### Bioinformatic analysis

Fosmids from β-galactosidase positive clones were prepared using the Qiagen plasmid preparation kit. Fosmid DNA was single- or double-digested with *Bam*HI and *Sma*I (Thermo Fisher Scientific) and the restriction products were visualised using agarose gel electrophoresis. Clones that had distinct restriction patterns were end sequenced (Eurofins genomics). The forward and the reverse nucleotide sequences generated from end sequencing reactions of clones were analysed using Blast (https://blast.ncbi.nlm.nih.gov/Blast.cgi). Conserved domain databases (CDD) and Pfam were used to identify the conserved protein regions. Smart BLAST and Constraint-Based Multiple Alignment Tool (COBALT) (Papadopoulos and Agarwala 2007) were used to identify homologues with the landmark sequences. In addition, PSORTb subcellular localisation tool was implemented to predict subcellular protein localisation (Yu et al. 2010). Homology-based structure prediction was done using SWISS-MODEL (https://swissmodel.expasy.org/).

### Cloning of identified β-galactosidase *bgaC* gene of *B. adolescentis*

The β-galactosidase *BAD_1582* (*bgaC*) gene sequence of *B. adolescentis* ATCC15703 was used to design cloning primers for PCR (CACCATGGCAGATACAGCCGAACTC and GAACAGCTTGAGCTGAACGTTGAG). With fosmid clone 31 as template, Velocity DNA polymerase (Bioline) was used to generate a blunt end product (25 cycles: 30 s at 98 °C, 30 s at 63 °C, 1 min and 30 s at 72 °C). PCR products were isolated by DNA gel purification using Promega Wizard R SV gel and PCR clean-up system following the manufacturer’s instructions. The purified blunt end DNA was cloned into expression plasmid pET101 (Invitrogen) following the directional cloning kit protocol. pET101 harbouring the *bgaC* gene was transformed into the expression host *E. coli* T7 express *lac*Z^−^ cells (Qin et al. 2010); the resulting clone was named *E. coli* T7express (pDMg1a). The primers ACGTATGCCTCGAATCG and CATATTTGGATAGCTC were used to determine the presence of *bgaC* in fosmids by PCR using Taq DNA polymerase (Bioline), followed by visualisation of the PCR products by agarose gel electrophoresis.

### Expression and purification of BAD_1582

*E. coli* T7 Express (pDMg1a) was grown to mid-log phase (OD_600nm_ between 0.4 and 0.7). Isopropyl-β-d-thiogalactopyranoside (IPTG, 1 mM) was added and the culture was incubated for 4 h at 37 °C to induce expression. An aliquot of the culture was then assayed for β-galactosidase activity using the Miller assay (Miller 1972). Cells were harvested by centrifugation and the pelleted cells were lysed with bug buster protein extraction reagent supplemented with 2 μl/ml lysozyme (Merck, USA). The lysate was centrifuged at 4 °C for 20 min at 16,000*g*, and the total cell lysate, the soluble fraction and the insoluble fractions were analysed by SDS-PAGE under denaturing condition to determine the localisation of the expressed enzyme. The soluble fraction containing the enzyme was then subjected to Ni^2+^-nitrilotriacetate (Ni^2+^-NTA) affinity chromatography to purify the His-tagged enzyme (Thermo Fisher) according to the manufacturer’s instruction.

The eluted fractions were concentrated and lower molecular mass proteins and imidazole removed using 100-kDa molecular weight cut-off (MWCO) Amicon Ultra-4 centrifugal filters (Merck, USA) and PBS buffer exchange. The retained protein (approximately > 100 kDa) concentration was determined by Bradford assay using bovine serum albumin (BSA) as standard. To determine the purity of the eluted fractions and the concentrated protein, SDS-PAGE was performed. After samples were electrophoresed on a 10% polyacrylamide gel, with broad range protein standards (11–245 kDa), the gels were stained both with Coomassie Brilliant blue R 250 and silver stain (Conway et al. 2016) for visualisation of protein. The concentrated protein was stored in aliquots at 4 °C for immediate use, while for long-term storage 50% (v/v) glycerol was added and stored at − 20 °C.

### Immunoblotting

Proteins were separated by SDS-PAGE and transferred to a nitrocellulose membrane. The nitrocellulose membrane was washed with PBS, blocked with 5% skim milk in PBS and probed using a monoclonal anti-polyhistidine antibody conjugated to horseradish peroxidase (1:2000 dilution) (Sigma). Visualisation was carried out by staining the nitrocellulose with tetramethylbenzidine (TMB) solution (Sigma) until sufficient colour was developed.

### β-Galactosidase activity assay

All β-galactosidase activity assays (both hydrolysis and transglycosylation) were conducted with three independent biological replicates and the data presented are the means of the three experiments.

β-Galactosidase activity of the BgaC protein was determined using the chromogenic substrate *ortho*-nitrophenyl-β-d-galactopyranoside (ONPG) or lactose. The assay for ONPG was carried out in microtiter plates containing 8.75 mg/ml purified enzyme, 2 mM ONPG in 50 mM sodium phosphate buffer in a final volume of 100 μl, at pH 7.0 and 37 °C for 30 min. The reaction was stopped by addition of sodium carbonate to a final concentration of 500 mM. The hydrolysis product *ortho-*nitrophenol was detected by measuring absorbance at 420 nm (Biotek microplate spectrophotometer). A unit of an enzyme (1 U) is defined as the amount of the enzyme required to liberate 1 μmol *o*NP from the ONPG substrate per minute in 50 mM sodium phosphate buffer (pH 7.0) at 37^o^C.

The assay for lactose hydrolysis was carried out with 89.5 μg/ml purified enzyme and 5 mM lactose in 50 mM sodium phosphate buffer at pH 7.0 and 37 °C for 30 min and the enzyme was then inactivated by incubating at 95 °C for 5 min. The released glucose was quantified using a glucose oxidase/peroxidase assay kit (Sigma) and measuring absorbance at 540 nm. A unit of enzyme (1 U) is defined as the amount of enzyme required to liberate 1 μmol glucose from lactose per minute in 50 mM sodium phosphate buffer at pH 7 and at 37 °C.

### Effects of pH, temperature, divalent cations and denaturants/detergents on the activity and stability of BAD_1582

The optimum pH of the enzyme was determined by using ONPG as a substrate in 50 mM sodium phosphate buffer at pH ranges of 4.0 to 10.0. The pH stability of the enzyme was determined by pre-incubating the enzyme in 50 mM sodium phosphate buffer set at pH 4.0–10.0 for 24 h at 4 °C. The pH of the pre-incubated enzyme was adjusted to pH 7.0 before the β-galactosidase activity was measured.

The effect of temperature on the enzyme activity was determined at different temperatures (from 0 to 60 °C) in 50 mM sodium phosphate buffer at pH 7.0. The temperature stability of the enzyme was determined by pre-incubating the enzyme in 50 mM sodium phosphate buffer at the selected temperatures for 1 h, after which the reactions were brought to 37 °C and β-galactosidase activity was determined.

The effect of metal ions on the activity of the enzyme was determined at pH 7.0. The effect of 10 mM K^+^ (KCl) and Mg^2+^ (MgCl_2_) on enzyme activity was determined in 50 mM sodium phosphate buffer. As most divalent cations precipitated in sodium phosphate buffer, CaCl_2_, MgCl_2_, ZnCl_2_, FeCl_3_, CuSO_4_ and MnCl_2_ at a final concentration of 10 mM were dissolved in 100 mM Tris-HCl buffer and enzyme activity determined in this buffer. To determine the effect of EDTA on the activity of BgaC, the enzyme was pre-incubated in 10 mM EDTA in 50 mM sodium phosphate buffer pH 7.0 for 3 h at 4 °C. Controls containing the same amount of enzyme incubated in sodium phosphate buffer containing 10 mM MgCl_2_ and enzyme in sodium phosphate buffer with no additional divalent metal ions were similarly prepared. Then, the β-galactosidase activity was determined.

The effects of detergents and denaturants on the activity of BgaC were determined by carrying out the β-galactosidase assay in 50 mM sodium phosphate buffer supplemented with 10 mM MgCl_2_ pH 7.0 and further supplemented with the following reagents: sodium dodecyl sulfate (0.1, 0.5 and 1%), urea (0.1, 0.5 and 1 M), Triton X-100 (0.1, 0.5 and 1%) and β-mercaptoethanol (1, 10 and 50 mM).

### Substrate specificity and kinetic parameters

To determine the substrate specificity of the enzyme, different chromogenic nitrophenyl–based substrates with α- and β-linked sugars were used. BgaC was incubated with the nitrophenyl analogues at a final concentration of 2 mM in 50 mM sodium phosphate buffer/10 mM MgCl_2_ at pH 7.0 and 37 °C and its hydrolytic activity was measured by absorbance at 420 nm. The nitrophenyl substrates used were *p*NP-β-d-galactopyranoside (*p*NPG), *p*NP-β-d-glucopyranoside (*p*NP-β-d-Glc), *p*NP-α-d-galactopyranoside (*p*NP-α-d-Gal), *p*NP-α-d-mannopyranoside (*p*NP-α-d-Man), *p*NP-α-L-fucopyranoside (*p*NP-α-l-Fuc) (Toronto Research Chemicals, TRC), *p*NP-α-l-xylopyranoside (pNP-α-l-Xyl) (TRC), *p*NP-2-acetamido-2-deoxy-β-d-galactopyranoside (*p*NP-β-d-GalNAc)(TRC) and *p*NP-2-acetamido-2-deoxy-β-d-glucopyranoside (*p*NP-GlcNAc) (Carbosynth) and ONPG.

The kinetic parameters of BgaC were determined by measuring activity at different concentrations of ONPG (0.25, 0.5, 0.75, 1, 2.5, 5, 10 and 20 mM) and lactose (5, 10, 25, 50, 100 and 200 mM) in 50 mM sodium phosphate buffer pH 7.0 at 37 °C in a time course discontinuous enzyme assay monitored for 20 min. Aliquots were withdrawn at 2-min intervals. For the ONPG assay, the aliquots were mixed with 500 mM sodium carbonate to stop the reaction and absorbance at 420 nm was recorded, and for the lactose assay aliquots were incubated at 95 °C for 5 min to inactivate the enzyme, and the glucose oxidase/peroxidase assay kit was used to determine the amount of glucose liberated. *V*_max_ and *K*_*m*_ were calculated using the enzyme kinetics module tool of SigmaPlot software version 14.0 (Systat Software, San Jose, CA) from initial velocity versus substrate concentration plot. Both Michaelis-Menten kinetics and Lineweaver-Burke plot transformation were implemented to determine *V*_max_ and *K*_*m*_.

### Inhibition assays using lactose, galactose and glucose

The β-galactosidase activity of BgaC was determined in the presence of lactose (0.25–75 mM), Gal and Glc (5–700 mM) as potential inhibitors and 1 mM ONPG as substrate. The *K*_*i*_ of lactose or Gal on BgaC activity was determined from the progress curves of the time course assay by applying competitive inhibition kinetics using Sigma Plot enzyme kinetic module.

### Transglycosylation activity assay

Transglycosylation assays were carried out using 1.5 units/ml BgaC enzyme in 50 mM sodium phosphate buffer/10 mM MgCl_2_ at 37 °C at pH 7.0 for 24 h. The first reaction set used *p*NPG as galactosyl donor and various sugars as acceptors: fructose (Fru), l-arabinose (Ara), GlcNAc, Gal, Glc and lactose. The assay contained 100 mM *p*NPG (25 mM *p*NPG when lactose was used as acceptor) and 200 mM acceptor sugar. In the second reaction set, 234 mM lactose was used both as a galactosyl donor and an acceptor. In the third reaction set, 200 mM lactose was used as a galactosyl donor and 100 mM l-fucose or *N*-acetylneuraminic acid (Neu5Ac) was used as an acceptor. After 24-h incubation, the enzyme was inactivated by heating at 95 °C for 5 min. Transglycosylation products were also monitored in a time-dependent assay, containing 234 mM lactose in 50 mM sodium phosphate buffer (pH 7.0) and 1.5 unit/ml enzyme at 37 °C for 72 h. Aliquot were withdrawn at 4, 8, 24, 48 and 72 h.

Samples were analysed using TLC with silica gel 60G F_254_ glass/aluminium foil plates as stationary phase. Samples and standards (1 μl) were spotted on the plates and the plates were immersed on a mobile phase of n-propanol:distilled water:ethyl acetate (7:2:1 v/v/v). The standard sugars were prepared in 50 mM sodium phosphate buffer (pH 7.0) at same concentration as in transglycosylation assays: GOS (Carbosynth), GOS (Teagasc) (8%), and lactose, Glc, Gal, Fru, L-Ara, GlcNAc, l-fucose, Neu5Ac and *p*NPG (75 mM). After separation, TLC plates were sprayed with 10% sulfuric acid in ethanol, allowed to completely dry and then charred at 105 °C for 5 min to visualise carbohydrates (so *p*NP would not be visualised with this method). *R*_*f*_ values for each spot of transglycosylation product were calculated by taking the ratio of distance travelled by each spot to distance travelled to solvent front and compared to the *R*_*f*_ values of the standard sugars.

### High-performance liquid chromatography

One milligram of each lyophilised sample obtained from transglycosylation reactions and 1 mg each of lactose and standard GOS were labelled with 50 μl labelling reagent per reaction (0.35 M 2-aminobenzamide (2-AB), 1 M sodium cyanoborohydride (NaCNBH_3_) dissolved in 30% acetic acid in DMSO) in amber tubes (Bigge et al. 1995). Labelling reactions were incubated in the dark at 65 °C for 2.5 h with gentle shaking at 200 rpm and samples were subsequently protected from light. Labelled samples were purified using Glycoclean S cartridges (ProZyme) following the manufacturer’s instructions and dried by centrifugal evaporation. Samples were reconstituted in 100 μl high-performance liquid chromatography (HPLC)–grade water and a 1:10 dilution of each sample was prepared. The diluted samples (10 μl) were injected onto a GlycoSep N-Plus HPLC column (PROzyme) equipped with a guard column on a Waters Alliance 2695 system (Waters, MA, USA) and separated at column temperature of 30 °C using gradient elution. The mobile phase consisted of HPLC-grade acetonitrile (A) and 50 mM ammonium formate, pH 4.4 (B), and the gradient started at 80% A to 47% A over 47.5 min, held at 47% for 1 min, changed to 0% A for 3 min to wash the column and then returned to 80% A over 1 min to re-equilibrate for 13 min before the next injection, all at a flow rate of 0.667 ml/min. Elution fluorescence was monitored at λ_ex_ 330 nm and λ_em_ 420 nm on a Waters 474 fluorescence detector.

### Statistical analysis

All experiments were conducted with three independent biological replicates and graphs were plotted from the mean of the three experiments. Error bars in figures represent the standard deviation (SD) (mean ± SD). Statistical significance was determined by Student’s *t* test, and significance was represented with *p* values < 0.001 = ***, < 0.01 = ** and < 0.05 = *.

## Results

### Construction of metagenomic library

A human faecal microbiome metagenomic library with insert sizes of 30–40 kb was constructed using the Copy Control-Fosmid Library Production system. Forty-two thousand distinct clones were generated for the human faecal microbiome metagenomic library. To assess the diversity of the library, 15 random clones were analysed by restriction pattern analysis and end sequencing of the fosmid inserts. Ten (67%) harboured different fragments of DNA originating from *B. adolescentis* both in the forward (5′ end of inserts) and reverse (3′ end of inserts) sequences with identities of 95–100% for most of the clones. However, clone 1 matched *B. adolescentis* with 77% identity and 95% identity for the forward and reverse sequences, respectively. One clone, clone 12, had a DNA fragment that originated from *Bacteriodes fragilis* (99% and 100% identity of forward and reverse sequence, respectively)*.* Two clones matched *Gordonibacter pamelaeae* with 80% identity (clone 10) and *Collinsella aerofaciens* with 94% identity (clone 13) on reverse and forward sequences, respectively, but no significant similarity was found on the forward and reverse sequences, respectively, of these two clones. On the other hand, clone 9 had similarity with a different bacterial species at each end of the insert. The forward sequence of clone 9 had similarity with *Bifidobacterium bifidum* (83% identity) while the reverse sequence had a match with *Olsenella uli* (73% identity). Thus, the clones were distinct amongst themselves and some of them had either low or no significant similarity in the database, indicating the origin of DNA from these clones could be from previously uncharacterised members of the microbiome. Having established the genetic diversity of the library, it was then used for functional screening for β-galactosidases.

### Functional screening for β-galactosidases in the human faecal microbiome metagenomic library

Sixteen thousand clones were screened for β-galactosidase activity using MacConkey lactose agar. Clones expressing active β-galactosidase appeared pink, as the acidic fermentation products from lactose utilisation changed the colour of the pH indicator within the agar. The control *lacZ*^*−*^ *E. coli* EPI300 (pCC1FOS) appeared colourless, as did clones without active β-galactosidase. Thirty-two β-galactosidase-positive clones were identified (Electronic Supplementary Material, Fig. S1a). When streaked individually on MacConkey lactose agar, they showed strong lactose hydrolysis, even compared to *lac*Z positive *E. coli* BL21 (DE3), whereas the negative control *E. coli* EPI300 (pCC1FOS) displayed no colour change (Electronic Supplementary Material, Fig. S1b)*.* β-Galactosidase activity was confirmed using LB agar supplemented with X-Gal for thirty clones, which formed characteristic blue colonies resulting from X-Gal hydrolysis. The positive control *E. coli* BL21 (DE3) also formed blue colonies while the negative control host strain did not (Electronic Supplementary Material, Fig. S1c).

Fosmids were prepared from each of the 30 β-galactosidase-positive clones and were characterised by restriction pattern to identify distinct clones. Seven distinct clones were identified from the comparison of restriction patterns and subsequent analyses focused on one representative of each. The Miller assay was conducted on these clones using ONPG as a substrate, and high β-galactosidase activity (600–5,795 Miller units) was detected, while the negative control *E. coli* EPI 300 (pCC1FOS) had low activity (36 Miller units).

### Bioinformatic analysis to identify β-galactosidase gene (BAD_1582) from human faecal microbiome metagenome clones

**Fig. 1:**
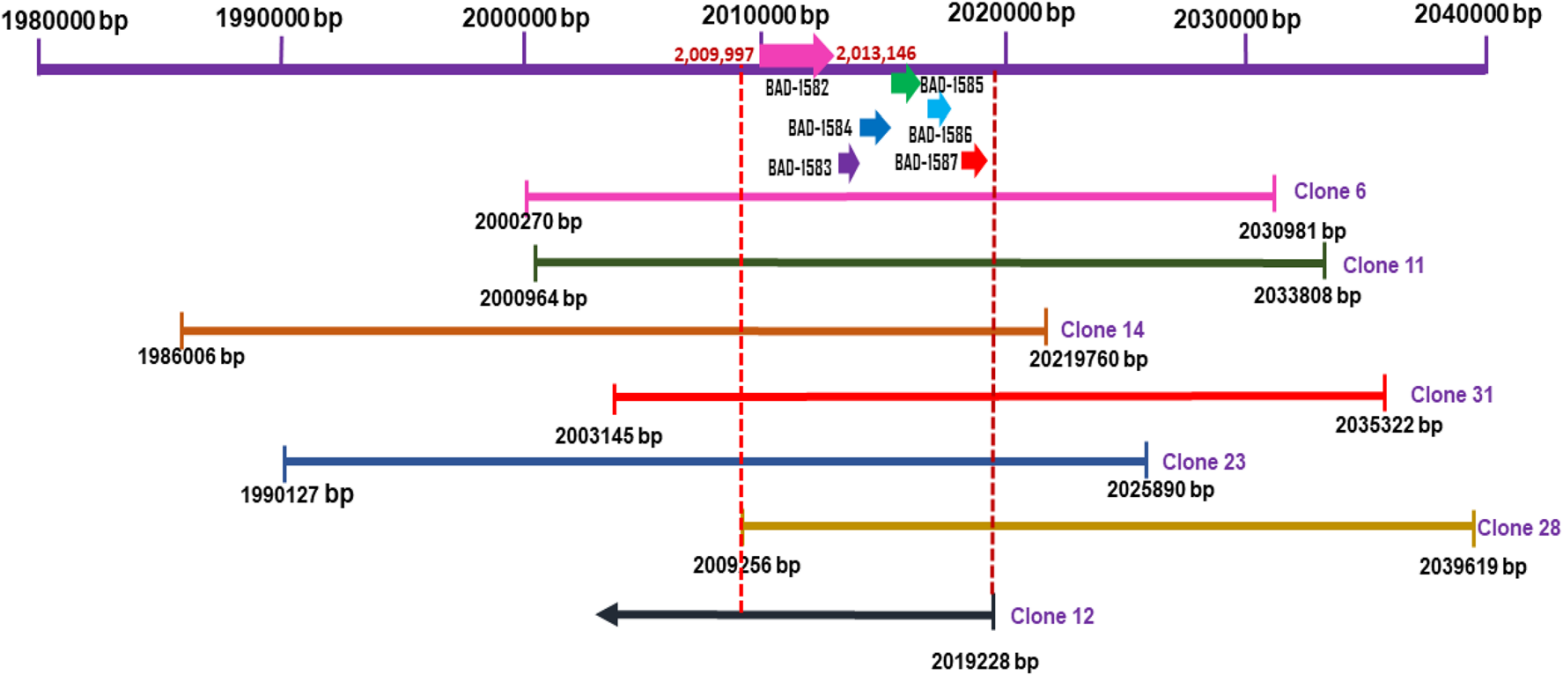
Gene map of fosmids which incorporated regions of the *B. adolescentis* genome and their six shared genes. These include the β-galactosidase gene (BAD_1582, *bgaC*), HdeD family acid-resistance protein (BAD_1583), LacI family transcriptional regulator (BAD_1584), carbohydrate ABC transporter substrate-binding protein (BAD_1585), sugar ABC transporter permease (BAD_1586), and carbohydrate ABC transporter permease (BAD_1587). The numbers at the start and end of each clone show location of the nucleotide sequences on the ATCC15703 genome

The seven distinct fosmids were sequenced to generate the 5′ and 3′ sequences of their inserts using primers complimentary to pCC1FOS. Nucleotide blast searches established the origin of the DNA and indicated that the fosmid sequences had high identity (98–99%) with genomic DNA of *B. adolescentis*, a common inhabitant of the adult human gut. One of these insert sequences had similarity with *B. adolescentis* only at the 3′ end and matched with an extrachromosomal DNA sequence (85% identity) at the 5′ end. The *B. adolescentis* strain from which this DNA fragment originated could have acquired the insertion sequences by horizontal transfer. The genome annotation of *B. adolescentis* ATCC15703 (NCBI) was used to identify possible β-galactosidase genes within the fosmid inserts. The gene map shows the regions of *B. adolescentis* genomic DNA contained within each fosmid (Fig. 1). All the clones shared the β-galactosidase *BAD_1582* gene and the adjacent five *BAD_1583* to *BAD_1587* genes. The adjacent genes encoded a sugar transporting permease, required by many bacteria to access sugars for adaptable growth in the gut environment. *BAD_1582*-specific PCR, using the fosmids from the thirty β-galactosidase-positive clones as template, demonstrated that all fosmids possessed *BAD_1582,* with the exception of clone 2 (Electronic Supplementary Material, Fig. S2a, b).

Through bioinformatic analysis, BAD_1582 was identified as a GH2 hydrolase with a homo-dimer structure. *Bifidobacterium* spp. protein sequences homologous to BAD_1582 (E-value = 0, % identity > 68%) and the 5 SmartBLAST reference landmark amino acid sequences for known GH2 β-galactosidases (E-value < e^−113^, % identity > 30%) were phylogenetically analysed. BAD_1582 is distantly related to GH2 β-galactosidases identified from different species of bacteria and plants: *Arabidopsis thaliana* (30% identity), soybean (32% identity) *Streptomyces coelicolor* (40% identity), *Thermotoga maritima* (33% identity), *E. coli* (32% identity). However, BAD_1582 is closely related to a β-galactosidase identified in several *Bifidobacterium* species: *B. ruminatium* (88% identity), *B. pseudocatenulatum* (77% identity), *Bifidobacterium* spp., N4GO5 (68% identity), *B. kashiwanohense* (68% identity), *B. tsurumiense* (68% identity), *B. catenulatum* (68% identity) and *B. longum* (68% identity), which indicates that these enzymes might be descended from the same ancestral protein (Electronic Supplementary Material, Fig. S3). In contrast, BAD_1582 has much lower homology to β-galactosidases from *B. animalis*, *B. infantis*, *B. bifidum*, *B. breve*, *B. dentium*, *B. reuteri* and other members of the *Bifidobacterium* taxon.

### Expression and purification of the β-galactosidase BAD_1582/BgaC

**Fig. 2:**
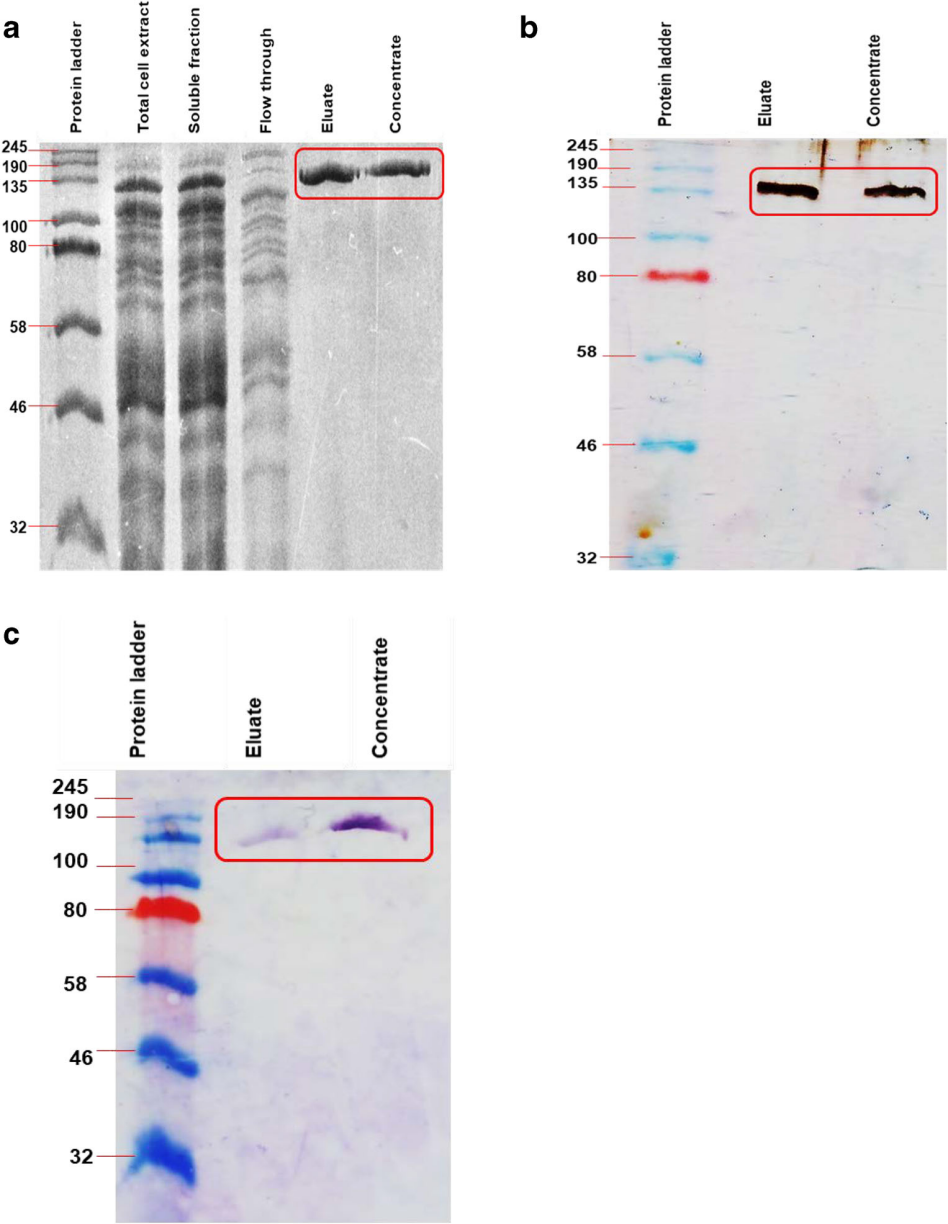
BgaC purification. Purification of the protein analysed by SDS-PAGE of purification step samples. Flow through and eluates were from Ni^2+^-NTA column purification and concentrate is after 100 kDa MWCO filtration. **a** Coomassie-stained gel, **b** silver-stained gel, and **c** immunoblot with anti-polyhistidine antibody conjugated to peroxidase and TMB staining

**Fig. 3:**
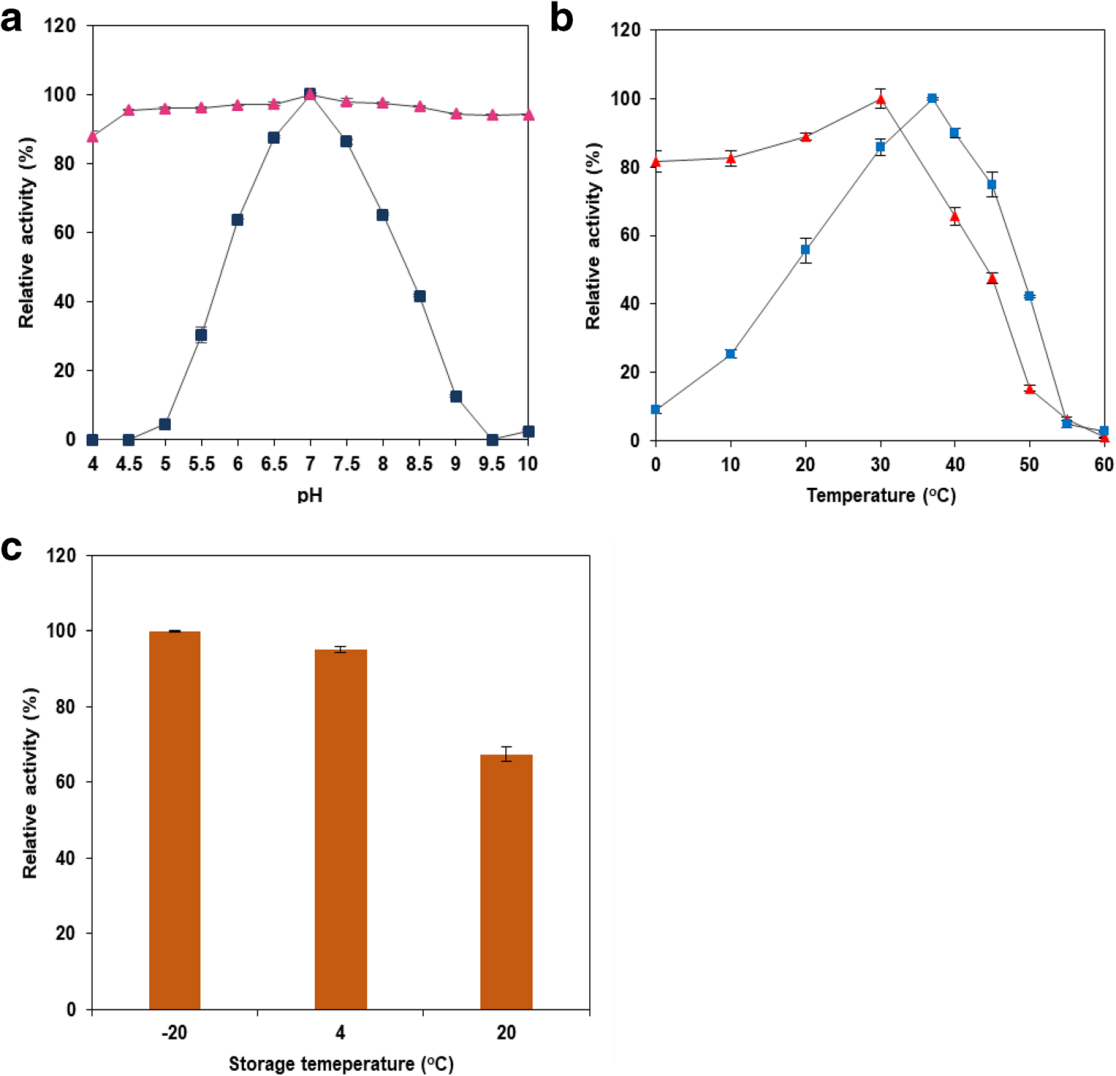
Effect of pH and temperature on BgaC. Effect of **a** pH and **b** temperature on β-galactosidase activity (squares) and stability (triangles) of BgaC in 50 mM sodium phosphate buffer, relative to activity at pH 7.0 and 37 °C, respectively. **c** Effect of storage temperature on BgaC activity, relative to activity after 4 °C storage. Values are the mean ± SD of three independent experiments

The β-galactosidase *BAD_1582* gene was cloned with a hexahistidine tag in the pET101 vector and transformed into the *lac*Z^-^ expression host *E. coli* T7 express. In order to express the recombinant BAD_1582, *E. coli* T7 express (pDMg1a) was cultured to exponential phase, 1 mM IPTG was added and induction of protein expression was continued for 4 h. After induction, a Miller assay carried out on IPTG-induced cells confirmed that enzyme activity was retained upon incorporation of the His-tag. The molecular mass of the His-tagged recombinant BgaC was predicted to be 116.5 kDa by amino acid content. IPTG-induced *E. coli* (pDMg1a) cells were harvested, and lysed, and the His-tagged enzyme was purified from the soluble fraction by Ni^2+^-NTA column chromatography. Remaining low molecular mass co-purified contaminant proteins were removed by filtration through a 100-kDa MWCO filter and the resulting approximately 120-kDa protein was the only protein present by Coomassie- and silver-stained SDS-PAGE analysis (Fig. 2a, b). Immunoblotting to detect the His-tag confirmed that the 120-kD purified protein was His-tagged BAD_1582 (Fig. 2c). The purified BAD_1582 was proven to have β-galactosidase activity with ONPG as substrate (Fig. 3a) and was consequently named BgaC, following the convention of the previous study of BgaB (BAD_1401) (Hinz et al. 2004).

### Biochemical properties of BgaC

β-Galactosidase activity of BgaC was tested over the pH ranges of 4.5 to 10.0 with ONPG as substrate. The enzyme had an optimum pH of 7.0 and retained 60% of its activity between pH 6.0 and 8.0 (Fig. 3a). BgaC was stable at all pH values tested, and 87% of its activity was retained at pH 4 (Fig. 3a).

The optimum temperature of this enzyme was determined to be 37 °C, but it was active over a wide range of temperatures, retaining 60% of its activity between 20 and 45 °C (Fig. 3b). The residual enzyme activity after 1 h incubation at those temperatures showed that the enzyme was active up to 40 °C (65% of its activity was retained) and its activity declined as temperatures increased (at 50 °C less than 20% activity retained) (Fig. 3b). Storage temperatures of 4 °C or −20 °C after 24-h incubation did not affect the activity of the enzyme. On the other hand, storage at room temperature (20 °C) for 24-h incubation decreased the activity of this enzyme by 35% (Fig. 3c). The enzyme was stored at 4 °C for more than 5 weeks and its activity was not altered (data not shown), but for long-term storage − 20 °C in a 50% glycerol stock was used and the activity was unaffected.

EDTA inhibited the activity of the enzyme which indicated a reliance on a divalent cation. The addition of Mg^2+^ enhanced the activity of BgaC by 30%, followed by Ca^2+^ and then Zn^2+^ (Table 1). On the other hand, activity was abolished by Cu^2+^ and showed 59% reduction in the presence of Mn^2+^ (Table 1).

**Table 1:** Effect of divalent cations and EDTA on BgaC activity

Substance (10 mM)	Relative activity (%) in phosphate buffer^a^	Relative activity (%) in 100 mM Tris HCl
KCl	105 ± 6	n.d
MgCl_2_	130 ± 6	174 ± 4
CaCl_2_	n.d	141 ± 4
ZnCl_2_	n.d	133 ± 5
MnCl_2_	n.d	41 ± 3
CuSO_4_	n.d	0 ± 0
EDTA	36 ± 1	n.d

Values are mean ± SD of three biological replicates, each with three technical replicates

^a^Relative to enzyme activity in absence of cations or EDTA

^b^*n.d* not determined

**Fig. 4:**
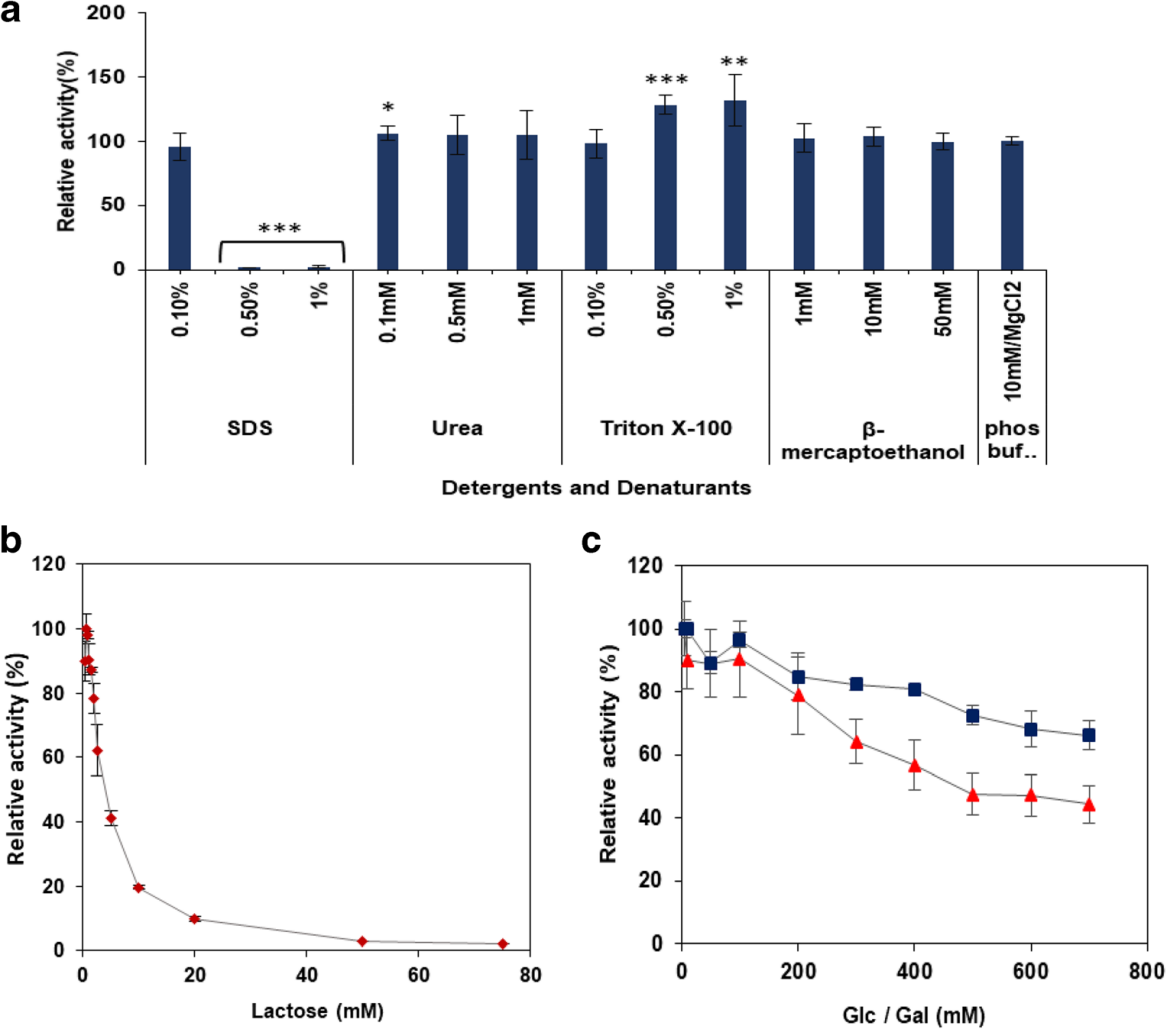
Inhibition of BgaC. **a** Effect of detergents and denaturants on BgaC activity in 50 mM sodium phosphate buffer. The data are relative to enzyme incubated in phosphate buffer/10 mM MgCl_2_ without detergent or denaturant. **b** Effect of lactose on BgaC activity on ONPG after 30-min incubation. **c** Effect of Glc (squares) and Gal (triangles) on BgaC activity on ONPG after 30-min incubation. Values are the mean ± SD of three independent experiments

As shown in Fig. 4a, the enzyme was severely inhibited by the addition of SDS at a concentration of 0.5% and above, whereas addition of urea at 0.1 mM enhanced the activity of BAD_1582, while no inhibition or enhancement were observed at concentrations of 0.5 mM and 1 mM. Triton X-100 enhanced activity at 0.5% and 1%, and no inhibition was observed at 0.1%. The presence of β-mercaptoethanol did not affect the activity of BgaC at the concentrations tested indicating the absence of disulphide bonds in the binding and catalytic region(s) of the enzyme.

### Substrate specificity and kinetic parameters

BgaC demonstrated high specific activity on *p*NPG and ONPG, with a higher specific activity towards *p*NPG (112 μmol/min/mg) compared to ONPG (18 μmol/min/mg). The specificity of BgaC was assessed and no activity was detected on α-linked Gal as in *p*NP-α-Gal. The enzyme did not hydrolyse any other tested *p*NP-based substrates, which included *p*NP-β-d-Glc, *p*NP-α-d-Man, *p*NP-α-l-Fuc, *p*NP-α-l-Xyl, *p*NP-β-GalNAc and *p*NP-β-GlcNAc, which demonstrates that this enzyme acted exclusively on terminal β-linked Gal residues.

β-Galactosidase activity was measured using different concentrations of ONPG (0.25–20 mM) and lactose (5–200 mM), and the enzyme rate (μmol min^−1^ mg^−1^) was calculated from non-linear regression fit model of substrate concentration versus enzyme rate, Michaelis-Menten plot (Electronic Supplementary Material, Fig. S4a, b). The enzyme had a *K*_*m*_ of 2.5 mM and *V*_max_ of 107 μmol min^−1^ mg^−1^ for ONPG and *K*_*m*_ of 3.7 mM and *V*_max_ of 22 μmol min^−1^ mg^−1^ for lactose. Besides, the catalytic efficiency, *K*_cat/_*K*_*m*_, of this enzyme for ONPG was 84 s^−1^ mM^−1^ which was 7-fold higher than that for lactose (12 s^−1^ mM^−1^) (Table 2). Nevertheless, the rate of hydrolysis of ONPG showed a substantial decrease with increasing concentration of the chromogenic substrate ONPG (≥ 2.5 mM), but the enzyme did not display substrate inhibition when lactose was used as substrate. The *K*_*i*_ of the enzyme for ONPG was 8 μM.

**Table 2:** Kinetic parameters of BgaC using ONPG and lactose as substrate, and in presence of lactose and Gal as competitive inhibitors of ONPG hydrolysis

Substrate/inhibitor	*K*_m_(mM)	*V*_max_ (μmol min^−1^ mg^−1^)	*K*_cat_ (s^−1^)	*K*_cat/_*K*_m_ (s^−1^ mM^−1^)	*K*_*i*_ (mM)
ONPG	2.5 ± 1.3	107 ± 43	209	84	0.0008 ± 0.0004
Lactose	3.7 ± 0.1	22 ± 1	43	12	n.d^a^
ONPG + lactose^b^	0.7 ± 0.004	129 ± 2	250	357	0.1 ± 0.003
ONPG + Gal	1.0 ± 0.004	20 ± 1	40	40	116 ± 5

^a^*n.d* no substrate inhibition observed and hence *K*_*i*_ was not determined for lactose

^b^ONPG (1 mM) was used as substrate, lactose (0.75–30 mM) and galactose (10–700 mM) were used as inhibitors

### Inhibition of BgaC by lactose, and tolerance to glucose and galactose

The effect of different concentrations of lactose (substrate) and its hydrolysis products, glucose and galactose, on the activity of BgaC was examined in the presence of the synthetic substrate ONPG (1 mM). β-Galactosidase activity towards ONPG declined markedly in the presence of increasing concentrations of lactose (Fig. 4b), suggesting that lactose is a competitive inhibitor to ONPG for binding to the enzyme active site. The *K*_*i*_ of lactose as a competitive inhibitor was 100 μM. In addition, *K*_*m*_, *V*_max_ and *K*_cat_ in the presence of lactose as a competitive inhibitor were 0.7 mM, 129 μmol min^−1^ mg^−1^ and 250 s^−1^, respectively (Table 2). Thus, the catalytic efficiency *K*_cat_/*K*_*m*_ towards ONPG was low in the presence of lactose.

The effects of the hydrolytic products of lactose on the activity of the enzyme towards ONPG were also determined. The enzyme was highly tolerant to glucose, as 66% of the enzyme activity was retained even in the presence of 700 mM glucose (Fig. 4c). However, it was observed that the enzyme was less tolerant to galactose and the activity of the enzyme decreased as the concentration of Gal increased above 100 mM (Fig. 4c). About 44% of its relative activity was retained at 700 mM Gal. The kinetic parameters of the enzyme towards ONPG in the presence of galactose as an inhibitor were determined. The *K*_*i*_, *K*_*m*_, *V*_max_ and *K*_cat_ for Gal were 116 mM, 1.0 mM, 20 μmol min^−1^ mg^−1^ and 40 s^−1^, respectively (Table 2). Having a relatively high *K*_*i*_ for Gal makes this enzyme an ideal candidate for its application in the enzymatic hydrolysis of lactose, due to its limited inhibition by its hydrolysis products.

### Transglycosylation activity and kinetics

**Fig. 5:**
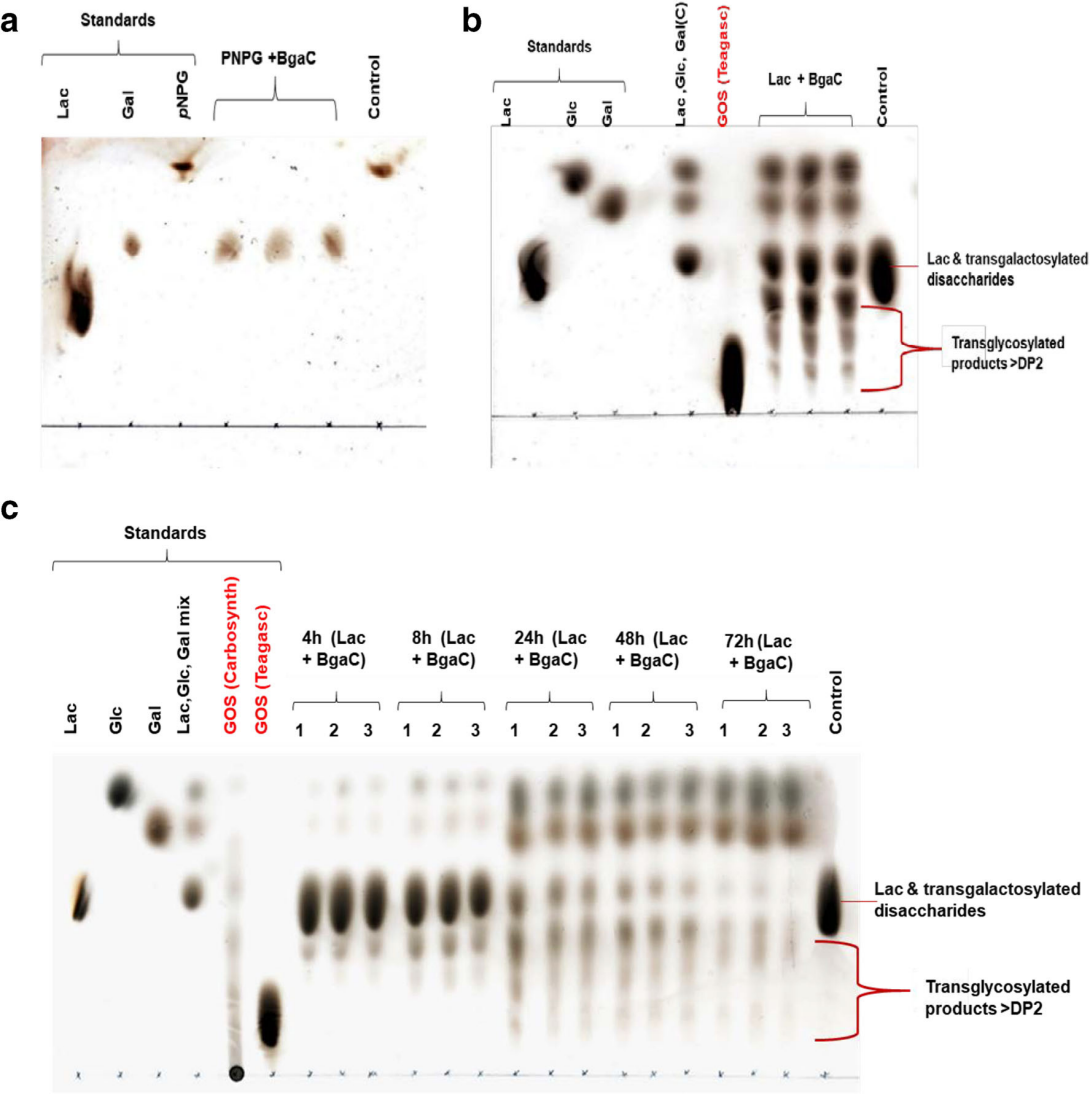
TLC analysis of transglycosylation and hydrolysis activities of BgaC. **a** Hydrolysis of *p*NPG after 24-h incubation. **b** Transglycosylation reaction products using lactose as donor and acceptor after 24-h incubation. **c** Kinetics of transglycosylation of BgaC using lactose as donor and acceptor, monitored between 4 and 72 h of incubation. Reactions with 1.5 unit/ml BgaC enzyme were performed and analysed in triplicate. Control reactions were incubated without enzyme. Reactions were performed at pH 7 and 37 °C

TLC was conducted to examine the hydrolysis and transglycosylation activity of BgaC. Gal was detected after cleavage of *p*NPG after 24-h incubation as expected for the hydrolytic function of the β-galactosidase (Fig. 5a). To assess the transglycosylation activity of BgaC, *p*NPG and lactose were used as galactosyl donors, and at 24-h transglycosylation occurred only in assays containing lactose as the donor and acceptor (Fig. 5b and Electronic Supplementary Material, Fig. S5 a-e and Fig. S6 a-c).

A kinetic study that examined products at incubation periods of 4, 8, 24, 48 and 72 h demonstrated that a lower degree of polymerisation (DP) of oligosaccharide reaction products, presumably GOS, started to accumulate after 4 h of incubation (Fig. 5c). Hydrolytic products were also detected at this time point at a somewhat lesser intensity, suggesting that shorter transglycosylation products are favoured at this time point. The DP of the transglycosylation products increased over time up to the third sampling point (24 h) and maintained that DP until the final sampling time. After 72-h incubation, there was little remaining lactose and the hydrolysis reaction was clearly favoured over transglycosylation as Glc and Gal were the most intense reaction products (Fig. 5c). These results indicate a balance of reactions with transglycosylation and hydrolysis both occurring simultaneously at all time points, but the balance favouring different reaction products shifting over time.

**Fig. 6:**
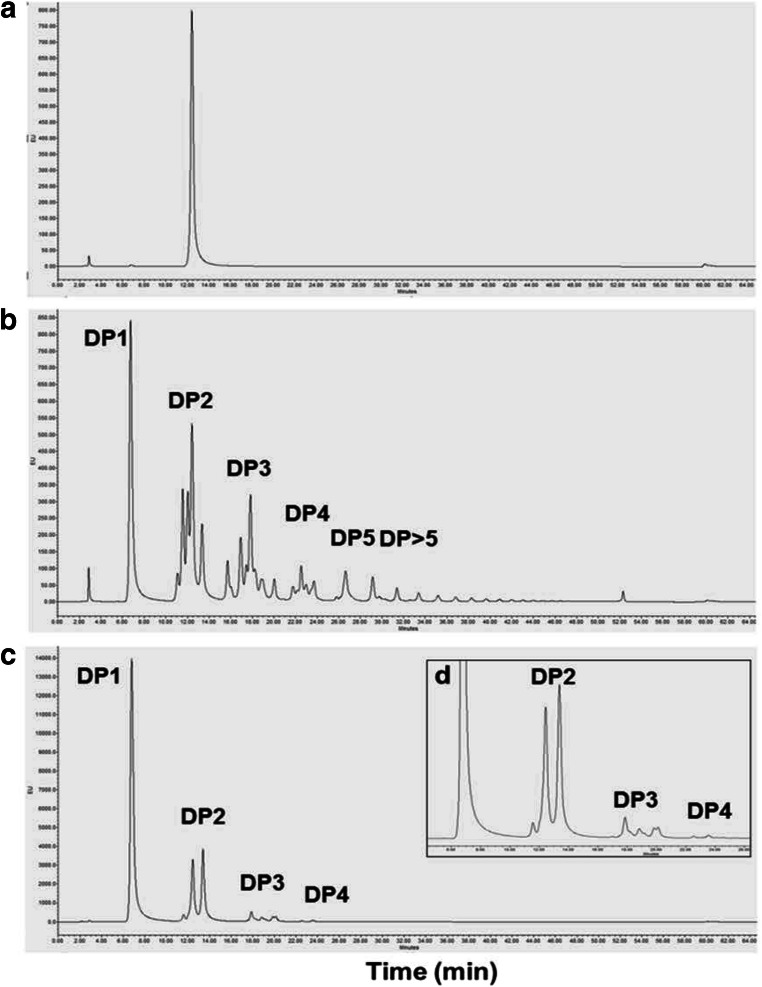
Chromatographs of transglycosylation and hydrolysis reactions of BgaC. (a) Chromatograph of lactose standard (DP2). (b) Chromatograph of GOS standard (DP1 to > 5). (c) BgaC reaction products after 24-h incubation, with reactants consisting of lactose (234 mM) both as donor and as acceptor incubated at 37 °C with 1.5 unit/ml enzyme at pH 7.0. (d) Inserted zoom of chromatograph (c) demonstrating BgaC transglycosylation products DP2 to DP4

HPLC analysis of the 2-AB labelled products of the transglycosylation reaction after 24 h confirmed that oligosaccharides (presumably galactooligosaccharides) with likely DP of 2 to 4 were produced by BgaC (Fig. 6(c, d)). Three individual trisaccharides (DP3) peaks and two distinct peaks of (DP4) were detected (Fig. 6(d)). Besides, three distinct disaccharides (DP2) were observed in Fig. 6(c), with the middle peak identified as lactose (compared to Fig. 6(a)). The other disaccharides produced were likely of different linkages compared to lactose and may have been of different monosaccharide composition (e.g. two Gal units compared to the Glc and Gal of lactose). The presence of multiple disaccharides in addition to the lactose reactant confirmed that BgaC could use not only lactose but also monosaccharides as acceptors for transglycosylation.

Reactions conducted using pNPG as the donor and either Gal or Glc as acceptors did not produce any transglycosylation products when analysed by TLC (Supplementary Figures S5a and S5b), so it is possible that the released pNP was inhibitory to transglycosylation in these reactions. On the other hand, it may be that some lactose (or other disaccharide produced) must be present to favour transglycosylation using a monosaccharide acceptor or, more likely, that these disaccharides were the hydrolysis products from the DP3 and DP4 transglycosylation products, as hydrolysis continually occurs during these reactions. Thus, the HPLC analysis supports the earlier suggestion of hydrolysis of transglycosylation products from the time course reaction observation by TLC analysis (Fig. 5c).

Therefore, the *B. adolescentis* BgaC β-galactosidase is a desirable enzyme for prebiotic manufacture and for application in dairy industries to produce lactose-free dairy products.

## Discussion

In this study, through function-based screening of a human faecal microbiome metagenome library, a new β-galactosidase enzyme from *B. adolescentis* was identified. BgaC possesses the industrially desirable properties of high substrate affinity for lactose and low product inhibition by Gal. Only a few β-galactosidases are used for food industry applications. The most widely used enzymes are obtained from *Kluveromyces* spp. and *Aspergillus* spp., as these source microbes are generally recognised as safe (GRAS) by the FDA (Adam et al. 2004). Even though these β-galactosidases have been widely utilised by dairy industries for removal of lactose and synthesis of GOS-based prebiotics, they have some limitations, such as low affinity for lactose and product inhibition by Gal (Erich et al. 2015). Thus, there is a demand for isolating new β-galactosidases that can surpass the aforementioned limitations.

Twenty-nine fosmids that had incorporated different regions of the genome of *B. adolescentis* harbouring *bgaC* were selected through our agar-based functional screening. The high abundance of *B. adolescentis* in the adult human gut microbiomes (Milani et al. 2015a) likely explains the selection of a high number of clones containing *bgaC*. The *bgaC* gene was identified as encoding a putative β-galactosidase during genome sequencing of this bacterium (Akiyama et al. 2015). Our phylogenetic analysis demonstrated that BgaC had strong evolutionary linkages with β-galactosidases from other species of *Bifidobacterium*, but these enzymes were not characterised. Some unrelated *Bifidobacterium* β-galactosidases have been isolated and characterised through expression of the recombinant enzyme in *E. coli*, and amongst these, a few displayed transglycosylation activity and synthesis of GOS (Arreola et al. 2014; Hsu et al. 2007; Oh et al. 2017; Yi et al. 2011).

*BAD_1582* is the first gene in a 6-gene sugar utilisation operon. *BAD_1583* encodes a protein of unknown function with a predicted cytoplasmic N-terminal region followed by 6 transmembrane domains that have some similarity with the HdeD acid-resistance protein and the DUF308_memb families. BAD_1584 is a LacI-family transcription regulator. The archetypic LacI repressor controls transcription of the *lac* operon by binding to the operator sequence in the absence of lactose (Oehler 2009). Downstream are genes encoding an ABC transporter substrate-binding protein (BAD_1585), sugar ABC transporter permease (BAD_1586) and carbohydrate ABC transporter permease (BAD_1587) which together form a complex to transport sugars (possibly lactose and other oligosaccharides) into the cell. In addition, the genome of *B. adolescentis* encodes two LacS symporters (BAD_1188 and BAD_1604), which probably have an equivalent function to the lactose permease of *E. coli*, transporting lactose and cations simultaneously.

An abundant repertoire of glycosidases enables *Bifidobacterium* to be amongst the most successful colonisers of the human gut, and in addition inter-species cross-feeding has been implicated in their success (De Vuyst and Leroy 2011; Egan et al. 2014; Milani et al. 2015b; Turroni et al. 2018). The genome of *B. adolescentis* ATCC15703 encodes eight galactosidase enzymes (https://www.ncbi.nlm.nih.gov/genome/proteins/683?genome_assembly_id=300284), of which two are α-galactosidases (BAD_RS08025/1528 (GH36) and BAD_RS08295/1576 (GH36)) and the remaining six are β-galactosidases (BAD_RS06400/1211 (GH42), BAD_RS07395/1401 (GH42) (Hinz et al. 2004; Van Laere et al. 2000), BAD_RS07400/1402, BAD_RS08435/1603 (GH42), BAD_RS08325/1582 (GH2) and BAD_RS08455/1605 (GH2)). Besides the galactosidases, the genome of *B. adolescentis* ATCC15703 encodes two *lacS* (lactose and galactose permease, GPH translocator family) genes (BAD_RS05925/1188 and BAD_RS08450/1604), which are symporters for translocating sugars and solutes. The presence of a high number of galactosidase enzymes in the genome of this bacterium supports the existence of possible specialisation amongst these enzymes for various glycan substrates, such as the host mucin oligosaccharides or dietary fibres.

Although six or more β-galactosidase genes are present in genomes of *B. adolescentis*, only *bgaC* was selected from the function-based screening of our metagenome library. Possible reasons the other *B. adolescentis* β-galactosidases were not detected could be because they were not expressed by the heterologous host *E. coli* due to factors such as codon bias, difference in transcription factors, poor expression of molecular chaperones, toxicity of proteins (Boel et al. 2016; Lipinszki et al. 2018; Rosano and Ceccarelli 2014) or because they were not translocated to their functional destination by the *E. coli* secretory system, e. g. extracellular proteins (Baneyx 1999). *B. adolescentis* grown in a culture supplemented with GOS induced the production of various galactosidases and BgaC was amongst the significantly upregulated enzymes (Akiyama et al. 2015; Van Laere et al. 2000). The LacI family transcription regulator *BAD_1584* gene present in each of the fosmid clones (Fig. 2) presumably encodes a regulator controlling the transcription of the *bgaC* operon (Oehler 2009), and thus BgaC was sufficiently expressed in the *E. coli* host.

Purified BgaC enzyme had optimal hydrolytic activity at pH 7.0 and 37 °C using ONPG as substrate. The hydrolytic activity of this enzyme at physiological pH and temperature is compatible with the bacterium’s environment in the human gut. On the other hand, *B. adolescentis* β-gal II (BAD_RS07395/1582, GH2) has an optimal pH of 6.0 and temperature of 35 °C using *p*NPG as a substrate (Hinz et al. 2004). Thus, the various *B. adolescentis* β-galactosidases display different properties. Temporary fluctuations from a fruit-rich diet, acidic secretions from the stomach or increased bile salts resulting from fermentation of dietary fibres by resident microbiota contribute to pH changes within the colon (Tomasello et al. 2016; Tomasello et al. 2014). The wide range of pH stability (pH 4–10) of BgaC would contribute to the pH adaptation of this beneficial bacterium.

BgaC displayed substrate specificity for β-linked Gal. Distinct flanking motifs in the active site of three GH42 β-galactosidase of *B. longum* affect their substrate specificities (Viborg et al. 2014). BgaC can hydrolyse lactose and *p*NPG efficiently even though the catalytic efficiency was lower than for ONPG. A couple of GH2 and GH42 β-galactosidases isolated from *Bifidobacterium* species and metagenome clones showed a higher catalytic activity towards ONPG than lactose (Arreola et al. 2014; Yi et al. 2011; Zhang et al. 2013). Lactose is a preferred substrate for some GH1 and GH2 β-galactosidases (Adam et al. 2004), while some GH35 and GH42 β-galactosidases act on other β-linked Gal containing glycosides. This specialisation for different substrates may explain why *B. adolescentis* possesses six β-galactosidases.

One of the important properties required in industrial enzymes is their tolerance to product inhibition. BgaC displayed high tolerance to galactose (*K*_*i*_ = 116 mM) and glucose (no inhibition detected). A couple of metagenome-derived β-galactosidases showing high tolerance to galactose with *K*_*i*_ values of 197 and 238 mM have been reported (Erich et al. 2015; Zhang et al. 2013). On the other hand, the hydrolytic activity of some β-galactosidases towards ONPG showed strong inhibition by galactose with low *K*_*i*_; for instance, *B. breve* β-gal I and II have *K*_*i*_ values of 15 and 34 mM, respectively (Arreola et al. 2014), and the *Aspergillus oryzae* β-galactosidase has a *K*_*i*_ of 25 mM (Vera et al. 2011). Having a high *K*_*i*_ for galactose makes BgaC an ideal candidate to undertake enzymatic hydrolysis of lactose with low risk of product inhibition.

BgaC exhibited transglycosylation activity at a relatively low lactose concentration (~ 200 mM) compared to known and commercial β-galactosidases that require high lactose concentrations (40% w/v or 1.17 M) (Guerrero et al. 2015; Hsu et al. 2007; Oh et al. 2017; Reuter et al. 1999). The ability of this enzyme to synthesise transglycosylation products, presumably GOS, could result in reduced GOS production costs using BgaC compared to currently available commercial enzymes. It is clear from the TLC and HPLC analysis that BgaC primarily produces oligosaccharides with DP2-4, exhibiting a narrower range and shorter length of oligosaccharide compared to commercial GOS. Depending on the prebiotic activity desired (e.g. certain beneficial bacterial species preferentially ferment oligosaccharides of specific lengths) (Garrido et al. 2013), this enzyme could be desirable to generate GOS with specific characteristics, either together with current enzymes or individually.

In the present work, the novel BgaC β-galactosidase enzyme from *B. adolescentis* was discovered from a human faecal microbiome metagenomic library. Its wide pH stability, stability at refrigeration storage conditions and high tolerance for Gal make it a desirable enzyme for the industrial application of removing lactose from dairy products. Furthermore, the transglycosylation activity of the enzyme demonstrates the potential for further utility in prebiotic manufacture. The successful discovery and expression of a functional β-galactosidase with valuable properties from a human faecal microbiome metagenomic library demonstrates that human *in vivo* microenvironments are a rich and important source of industrially useful enzymes.

## Supplementary Information

ESM 1 (PDF 600 kb)

## Data Availability

Data and material for this article are available upon request.

## References

[CR1] Adam AC, Rubio-Texeira M, Polaina J (2004). Lactose: the milk sugar from a biotechnological perspective. Crit Rev Food Sci Nutr.

[CR2] Agbavwe C (2017) Identification and characterisation of novel glycan binding bacterial adhesins encoded by the human gut microbial metagenome. PhD thesis, National University of Ireland, Galway

[CR3] Akiyama T, Kimura K, Hatano H (2015). Diverse galactooligosaccharides consumption by bifidobacteria: implications of β-galactosidase-LacS operon. Biosci Biotechnol Biochem.

[CR4] Arreola SL, Intanon M, Suljic J, Kittl R, Pham NH, Kosma P, Haltrich D, Nguyen T-H (2014). Two β-Galactosidases from the human isolate *Bifidobacterium breve* DSM 20213: molecular cloning and expression, biochemical characterization and synthesis of galactooligosaccharides. PLoS One.

[CR5] Baneyx F (1999). Recombinant protein expression in *Escherichia coli*. Curr Opin Biotechnol.

[CR6] Bigge JC, Patel TP, Bruce JA, Goulding PN, Charles SM, Parekh RB (1995). Nonselective and efficient fluorescent labeling of glycans using 2-amino benzamide and anthranilic acid. Anal Biochem.

[CR7] Boel G, Letso R, Neely H, Price WN, Wong KH, Su M, Luff J, Valecha M, Everett JK, Acton TB, Xiao R, Montelione GT, Aalberts DP, Hunt JF (2016). Codon influence on protein expression in *E. coli* correlates with mRNA levels. Nature.

[CR8] Cecchini DA, Laville E, Laguerre S, Robe P, Leclerc M, Doré J, Henrissat B, Remaud-Siméon M, Monsan P, Potocki-Véronèse G (2013). Functional metagenomics reveals novel pathways of prebiotic breakdown by human gut bacteria. PLoS One.

[CR9] Cheng J, Romantsov T, Engel K, Doxey AC, Rose DR, Neufeld JD, Charles TC (2017). Functional metagenomics reveals novel β-galactosidases not predictable from gene sequences. PLoS One.

[CR10] Conway JM, Pierce WS, Le JH, Harper GW, Wright JH, Tucker AL, Zurawski JV, Lee LL, Blumer-Schuette SE, Kelly RM (2016). Multidomain, surface layer-associated glycoside hydrolases contribute to plant polysaccharide degradation by caldicellulosiruptor species. J Biol Chem.

[CR11] De Vuyst L, Leroy F (2011). Cross-feeding between bifidobacteria and butyrate-producing colon bacteria explains bifdobacterial competitiveness, butyrate production, and gas production. Int J Food Microbiol.

[CR12] Egan M, O’Connell Motherway M, Kilcoyne M, Kane M, Joshi L, Ventura M, van Sinderen D (2014). Cross-feeding by *Bifidobacterium breve* UCC2003 during co-cultivation with *Bifidobacterium bifidum* PRL2010 in a mucin-based medium. BMC Microbiol.

[CR13] Erich S, Kuschel B, Schwarz T, Ewert J, Bohmer N, Niehaus F, Eck J, Lutz-Wahl S, Stressler T, Fischer L (2015). Novel high-performance metagenome β-galactosidases for lactose hydrolysis in the dairy industry. J Biotechnol.

[CR14] Ford T, Rickwood D (1982). Formation of isotonic nycodenz gradients for cell separations. Anal Biochem.

[CR15] Gänzle MG, Haase G, Jelen P (2008). Lactose: crystallization, hydrolysis and value-added derivatives. Int Dairy J.

[CR16] Garrido D, Ruiz-Moyano S, Jimenez-Espinoza R, Eom H-J, Block DE, Mills DA (2013). Utilization of galactooligosaccharides by *Bifidobacterium longum* subsp. *infantis* isolates. Food Microbiol.

[CR17] Gibson GR, Roberfroid MB (1995). Dietary modulation of the human colonic microbiota: introducing the concept of prebiotics. J Nutr.

[CR18] Guerrero C, Vera C, Conejeros R, Illanes A (2015). Transgalactosylation and hydrolytic activities of commercial preparations of β-galactosidase for the synthesis of prebiotic carbohydrates. Enzym Microb Technol.

[CR19] Hinz SW, van den Brock LA, Beldman G, Vincken JP, Voragen AG (2004). β-galactosidase from *Bifidobacterium adolescentis* DSM20083 prefers β (1,4)-galactosides over lactose. Appl Microbiol Biotechnol.

[CR20] Hsu C-A, Lee S-L, Chou C-C (2007). Enzymatic production of galactooligosaccharides by β-galactosidase from *Bifidobacterium longum* BCRC 15708. J Agric Food Chem.

[CR21] Kolida S, Gibson GR (2011). Synbiotics in health and disease. Annu Rev Food Sci Technol.

[CR22] Lee SW, Won K, Lim HK, Kim JC, Choi GJ, Cho KY (2004). Screening for novel lipolytic enzymes from uncultured soil microorganisms. Appl Microbiol Biotechnol.

[CR23] Lipinszki Z, Vernyik V, Farago N, Sari T, Puskas LG, Blattner FR, Posfai G, Gyorfy Z (2018). Enhancing the translational capacity of *E. coli* by resolving the codon bias. ACS Synth Biol.

[CR24] Liu P, Wang W, Zhao J, Wei D (2019). Screening novel β-galactosidases from a sequence-based metagenome and characterization of an alkaline β-galactosidase for the enzymatic synthesis of galactooligosaccharides. Protein Expr Purif.

[CR25] Lombard V, Golaconda Ramulu H, Drula E, Coutinho PM, Henrissat B (2014). The carbohydrate-active enzymes database (CAZy) in 2013. Nucleic Acids Res.

[CR26] Mawson AJ (1994). Bioconversions for whey utilization and waste abatement. Bioresour Technol.

[CR27] Milani C, Lugli GA, Duranti S, Turroni F, Mancabelli L, Ferrario C, Mangifesta M, Hevia A, Viappiani A, Scholz M, Arioli S, Sanchez B, Lane J, Ward DV, Hickey R, Mora D, Segata N, Margolles A, van Sinderen D, Ventura M (2015). Bifidobacteria exhibit social behavior through carbohydrate resource sharing in the gut. Sci Rep.

[CR28] Milani C, Mancabelli L, Lugli GA, Duranti S, Turroni F, Ferrario C, Mangifesta M, Viappiani A, Ferretti P, Gorfer V, Tett A, Segata N, van Sinderen D, Ventura M (2015). Exploring vertical transmission of bifidobacteria from mother to child. Appl Environ Microbiol.

[CR29] Miller J (1972). Experiments in molecular genetics.

[CR30] Neveu J, Regeard C, DuBow MS (2011). Isolation and characterization of two serine proteases from metagenomic libraries of the Gobi and Death Valley deserts. Appl Microbiol Biotechnol.

[CR31] Oehler S (2009). Feedback regulation of Lac repressor expression in *Escherichia coli*. J Bacteriol.

[CR32] Oh SY, Youn SY, Park MS, Kim HG, Baek NI, Li Z, Ji GE (2017). Synthesis of β-galactooligosaccharide using bifidobacterial β-galactosidase purified from recombinant *Escherichia coli*. J Microbiol Biotechnol.

[CR33] Papadopoulos JS, Agarwala R (2007). COBALT: constraint-based alignment tool for multiple protein sequences. Bioinformatics (Oxford, England).

[CR34] Qin J, Li R, Raes J, Arumugam M, Burgdorf KS, Manichanh C, Nielsen T, Pons N, Levenez F, Yamada T, Mende DR, Li J, Xu J, Li S, Li D, Cao J, Wang B, Liang H, Zheng H, Xie Y, Tap J, Lepage P, Bertalan M, Batto JM, Hansen T, Le Paslier D, Linneberg A, Nielsen HB, Pelletier E, Renault P, Sicheritz-Ponten T, Turner K, Zhu H, Yu C, Li S, Jian M, Zhou Y, Li Y, Zhang X, Li S, Qin N, Yang H, Wang J, Brunak S, Dore J, Guarner F, Kristiansen K, Pedersen O, Parkhill J, Weissenbach J, Bork P, Ehrlich SD, Wang J (2010). A human gut microbial gene catalogue established by metagenomic sequencing. Nature.

[CR35] Reuter S, Rusborg Nygaard A, Zimmermann W (1999). β-Galactooligosaccharide synthesis with β-galactosidases from *Sulfolobus solfataricus*, *Aspergillus oryzae,* and *Escherichia coli*. Enzym Microb Technol.

[CR36] Rosano GL, Ceccarelli EA (2014). Recombinant protein expression in *Escherichia coli*: advances and challenges. Front Microbiol.

[CR37] Simon C, Daniel R (2011). Metagenomic analyses: past and future trends. Appl Environ Microbiol.

[CR38] Tasse L, Bercovici J, Pizzut-Serin S, Robe P, Tap J, Klopp C, Cantarel BL, Coutinho PM, Henrissat B, Leclerc M, Dore J, Monsan P, Remaud-Simeon M, Potocki-Veronese G (2010). Functional metagenomics to mine the human gut microbiome for dietary fiber catabolic enzymes. Genome Res.

[CR39] Tomasello G, Tralongo P, Damiani P, Sinagra E, Di Trapani B, Zeenny MN, Hussein IH, Jurjus A, Leone A (2014). Dismicrobism in inflammatory bowel disease and colorectal cancer: changes in response of colocytes. World J Gastroenterol.

[CR40] Tomasello G, Mazzola M, Leone A, Sinagra E, Zummo G, Farina F, Damiani P, Cappello F, Gerges Geagea A, Jurjus A, Bou Assi T, Messina M, Carini F (2016). Nutrition, oxidative stress and intestinal dysbiosis: influence of diet on gut microbiota in inflammatory bowel diseases. Biomed Pap Med Fac Univ Palacky Olomouc Czech Repub.

[CR41] Torres DPM, Gonçalves MPF, Teixeira JA, Rodrigues LR (2010). Galactooligosaccharides: production, properties, applications and significance as prebiotics. Compr Rev Food Sci Food Saf.

[CR42] Turroni F, Milani C, Duranti S, Mahony J, van Sinderen D, Ventura M (2018). Glycan utilization and cross-feeding activities by bifidobacteria. Trends Microbiol.

[CR43] Uchiyama T, Miyazaki K, Yaoi K (2013). Characterization of a novel β-glucosidase from a compost microbial metagenome with strong transglycosylation activity. J Biol Chem.

[CR44] Van Laere KMA, Schols T, Beldman HA, Voragen AG (2000). Characterization of a novel β-galactosidase from *Bifidobacterium adolescentis* DSM 20083 active towards transgalactooligosaccharides. Appl Environ Microbiol.

[CR45] Vandenplas Y (2015) Lactose intolerance. Asia Pac J Clin Nutr 24(Suppl 1):S9–S13. 10.6133/apjcn.2015.24.s1.02 10.6133/apjcn.2015.24.s1.02 26715083

[CR46] Vera C, Guerrero C, Illanes A (2011). Determination of the transgalactosylation activity of *Aspergillus oryzae* β-galactosidase: effect of pH, temperature, and galactose and glucose concentrations. Carbohydr Res.

[CR47] Viborg AH, Katayama T, Abou Hachem M, Andersen MC, Nishimoto M, Clausen MH, Urashima T, Svensson B, Kitaoka M (2014). Distinct substrate specificities of three glycoside hydrolase family 42 β-galactosidases from *Bifidobacterium longum* subsp. *infantis* ATCC 15697. Glycobiology.

[CR48] Walton GE, van den Heuvel EG, Kosters MH, Rastall RA, Tuohy KM, Gibson GR (2012). A randomised crossover study investigating the effects of galacto-oligosaccharides on the faecal microbiota in men and women over 50 years of age. Br J Nutr.

[CR49] Wierzbicka-Woś A, Bartasun P, Cieśliński H, Kur J (2013). Cloning and characterization of a novel cold-active glycoside hydrolase family 1 enzyme with β-glucosidase, β-fucosidase and β-galactosidase activities. BMC Biotechnol.

[CR50] Yi SH, Alli I, Park KH, Lee B (2011). Overexpression and characterization of a novel transgalactosylic and hydrolytic β-galactosidase from a human isolate *Bifidobacterium breve* B24. New Biotechnol.

[CR51] Yu NY, Wagner JR, Laird MR, Melli G, Rey S, Lo R, Dao P, Sahinalp SC, Ester M, Foster LJ, Brinkman FS (2010). PSORTb 3.0: improved protein subcellular localization prediction with refined localization subcategories and predictive capabilities for all prokaryotes. Bioinformatics (Oxford, England).

[CR52] Zhang X, Li H, Li CJ, Ma T, Li G, Liu YH (2013). Metagenomic approach for the isolation of a thermostable β-galactosidase with high tolerance of galactose and glucose from soil samples of Turpan Basin. BMC Microbiol.

