# Supplementary material for: Metagenomic identification, purification and characterisation of the Bifidobacterium adolescentis BgaC β-galactosidase
Source: Appl Microbiol Biotechnol. 2021 Jan 11;105(3):1063–78. doi: 10.1007/s00253-020-11084-y (PMC7843569; doi:10.1007/s00253-020-11084-y)
Supplement: Supplementary file 1 — (PDF 600 kb) [file 253_2020_11084_MOESM1_ESM.pdf]

Journal name: Applied Microbiology and Biotechnology

Metagenomic identification, purification and characterization of the *Bifidobacterium adolescentis* BgaC β-galactosidase

Daniel Mehabie Mulualem[1], Christy Agbavwe[1], Lesley A Ogilvie[2], Brian V Jones[3], Michelle Kilcoyne[1], Conor O'Byrne[1], Aoife Boyd[1*]

[1]Discipline of Microbiology, School of Natural Sciences, National University of Ireland Galway, Galway, Ireland
[2]Max Planck Institute for Molecular Genetics, Berlin, Germany
[3]Department of Biology & Biochemistry, University of Bath, Bath, United Kingdom

* Corresponding author

E-mail: aoife.boyd@nuigalway.ie

Tel: +353 91 492404

Aoife Boyd ORCID ID: 0000-0002-4631-9792

Electronic Supplementary Material, figures and tables

Electronic Supplementary Material figure legends

Fig. S1 Selection of β-galactosidase expressing clones from human gut microbiome metagenome library. (**a**) β-galactosidase-positive clone with pink colony morphology. EPI300(pCC1FOS) (I), BL21(DE3) (II), Clone 11 (III) and Clone 31 (IV) on (**b**) MacConkey lactose agar and (**c**) X-Gal-supplemented LB agar.

Fig. S2 PCR of representative *BAD_1582*-harboring metagenome clones. PCR amplification of the target gene (3.15 kb) in (**a**) Clones 1-19 and (**b**) Clones 20-32. Clone 2 does not contain *BAD_1582*.

Fig. S3 Evolutionary relationship of BgaC with homologous proteins and landmark similar sequences. The evolutionary history was infered using UPGMA method. The optimal tree with the sum of branch length=3.18 is shown. The tree is drawn to scale, with branch lengths in the same units as those of the evolutionary distances used to infer the phylogenetic tree. The evolutionary distances were computed using the Poisson correction method and are in the units of the number of amino acid substitutions per site. The analysis involved 15 amino acid sequences. All positions containing gaps and missing data were eliminated. There were a total of 891 positions in the final data set. Evolutionary analyses were conducted in Mega 7. The top three sequences are BgaC of *B. adolescentis* ATCC 15703, L2-32 (99.8% identity) and AF14-56 (93.4% identity), representing the similarity range for this protein across the species. β-Galactosidases from *Bifidobacterium* species with identities ≥68% to BgaC were also incorporated in this analysis.

Fig. S4 Non-linear regression fit for calculation of the kinetic parameters of BgaC with **(a)** ONPG and **(b)** lactose as substrate.

Fig. S5 TLC analysis of BgaC transglycosylation and hydrolysis products **a-f** Transglycosylation reaction with *p*NPG as a donor and Glc **(a)**, Gal **(b)**, Frc **(c)**, L-Ara **(d)** and GlcNAc **(e)** as acceptors after 24 h incubation with 1.5 unit/ml enzyme and control without enzyme.

Fig. S6 Transglycosylation reaction products with *p*NPG as a donor and lactose as acceptor (technical triplicate, lanes 6-8) and transglycosylation reaction products with lactose both as donor and acceptor (technical triplicate, lanes 10-12) after 24 h incubation with 1.5 unit/ml enzyme and a control without enzyme (**a**). Transglycosylation reaction with lactose as donor and Fuc **(b)** and Neu5Ac **(c)** as acceptors after 24 h incubation with 1.5 unit/ml enzyme and a control without enzyme. All reactions were carried out in 50 mM phosphate buffer at pH 7 and at 37°C.

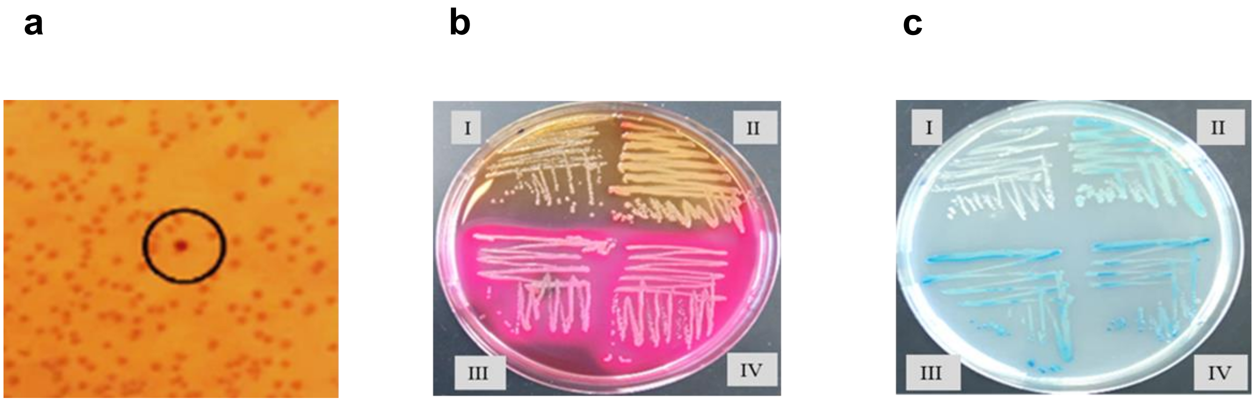
a
b
c
I
II
III
IV
I
II
III
IV

Fig. S1

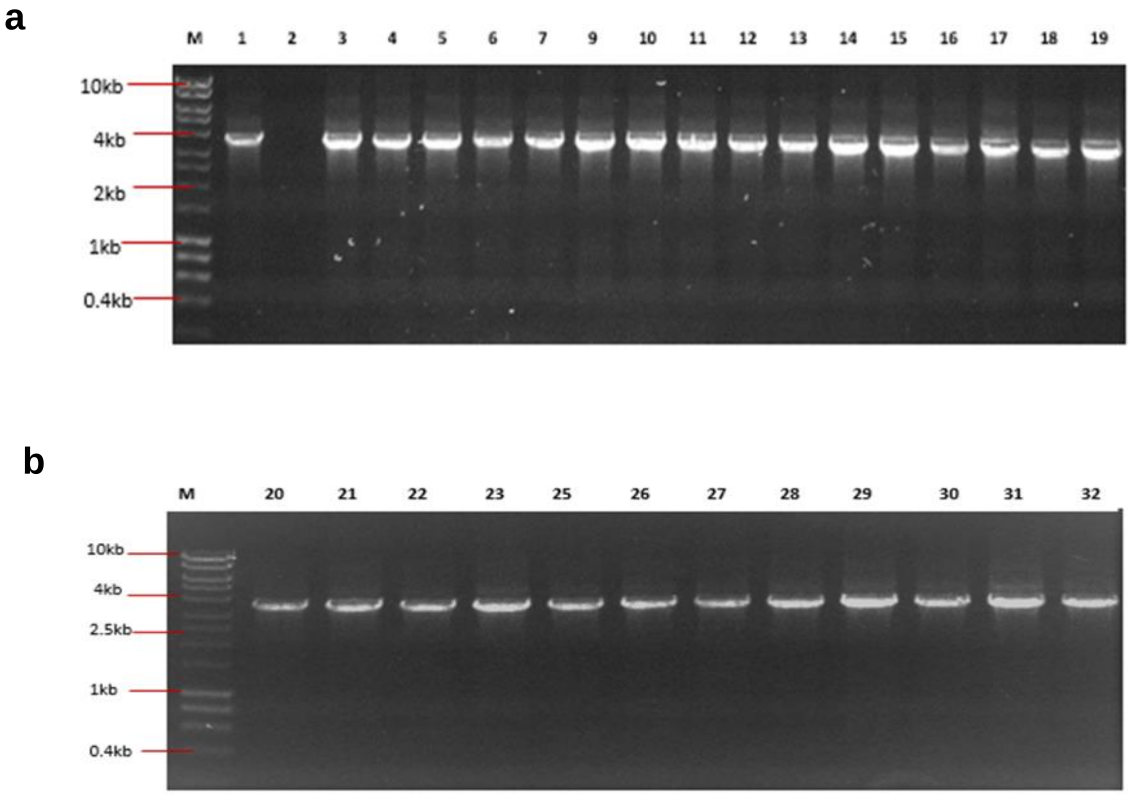
a
M 1 2 3 4 5 6 7 9 10 11 12 13 14 15 16 17 18 19
10kb
4kb
2kb
1kb
0.4kb
b
M 20 21 22 23 25 26 27 28 29 30 31 32
10kb
4kb
2.5kb
1kb
0.4kb

Fig. S2

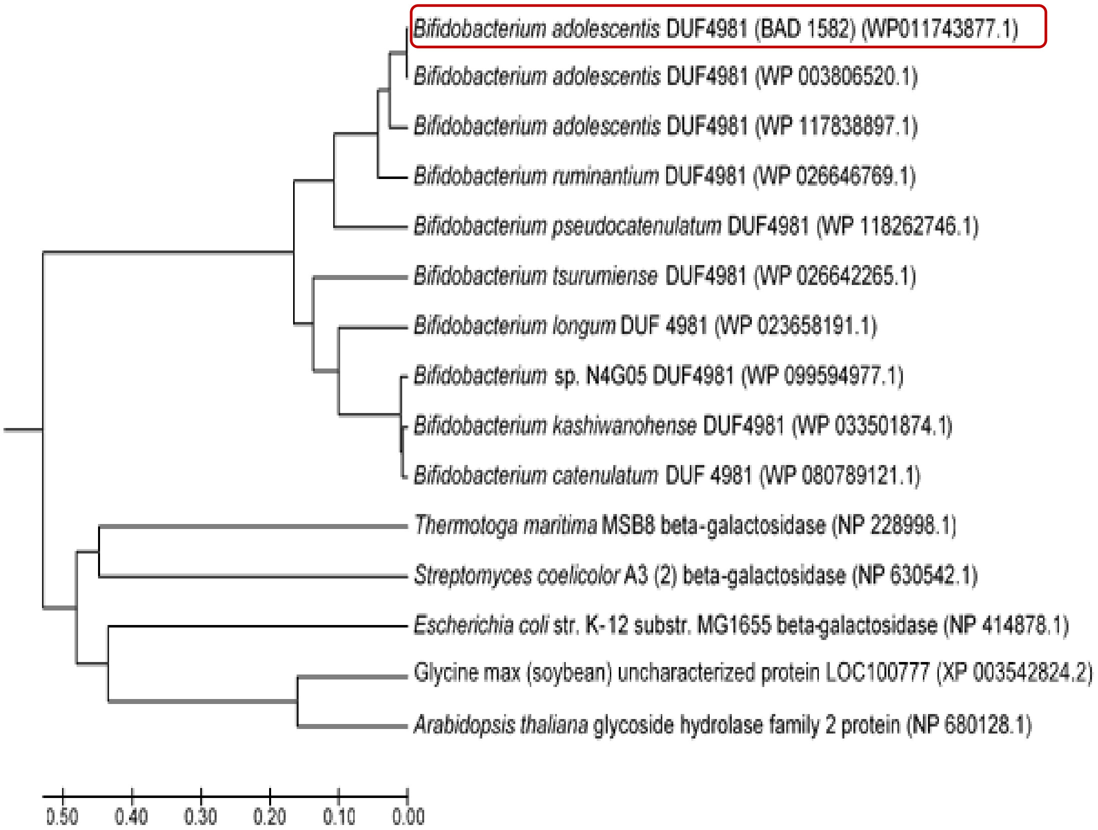

Bifidobacterium adolescentis DUF4981 (BAD 1582) (WP011743877.1)
Bifidobacterium adolescentis DUF4981 (WP 003806520.1)
Bifidobacterium adolescentis DUF4981 (WP 117838897.1)
Bifidobacterium ruminantium DUF4981 (WP 026646769.1)
Bifidobacterium pseudocatenulatum DUF4981 (WP 118262746.1)
Bifidobacterium tsurumiense DUF4981 (WP 026642265.1)
Bifidobacterium longum DUF 4981 (WP 023658191.1)
Bifidobacterium sp. N4G05 DUF4981 (WP 099594977.1)
Bifidobacterium kashiwanohense DUF4981 (WP 033501874.1)
Bifidobacterium catenulatum DUF 4981 (WP 080789121.1)
Thermotoga maritima MSB8 beta-galactosidase (NP 228998.1)
Streptomyces coelicolor A3 (2) beta-galactosidase (NP 630542.1)
Escherichia coli str. K-12 substr. MG1655 beta-galactosidase (NP 414878.1)
Glycine max (soybean) uncharacterized protein LOC100777 (XP 003542824.2)
Arabidopsis thaliana glycoside hydrolase family 2 protein (NP 680128.1)
0.50
0.40
0.30
0.20
0.10
0.00

Fig. S3

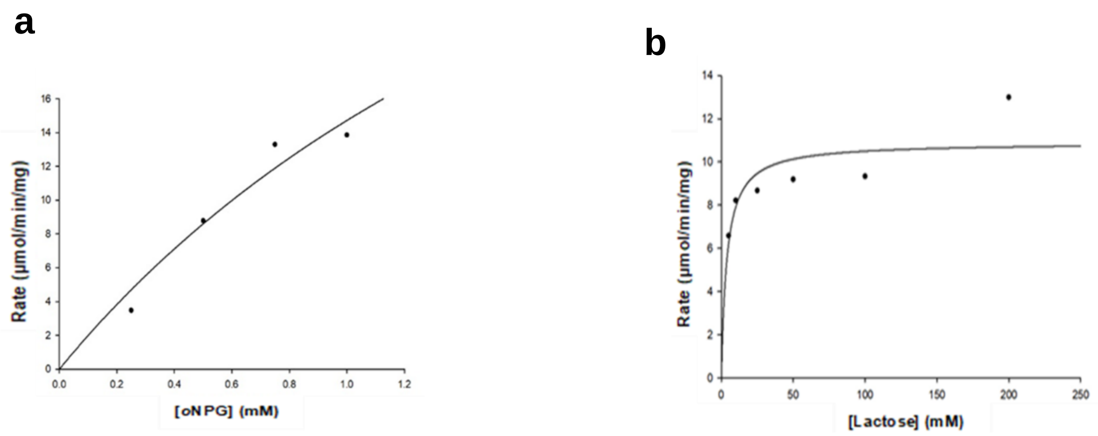
a
b
Rate (µmol/min/mg)
[oNPG] (mM)
[Lactose] (mM)

Fig. S4

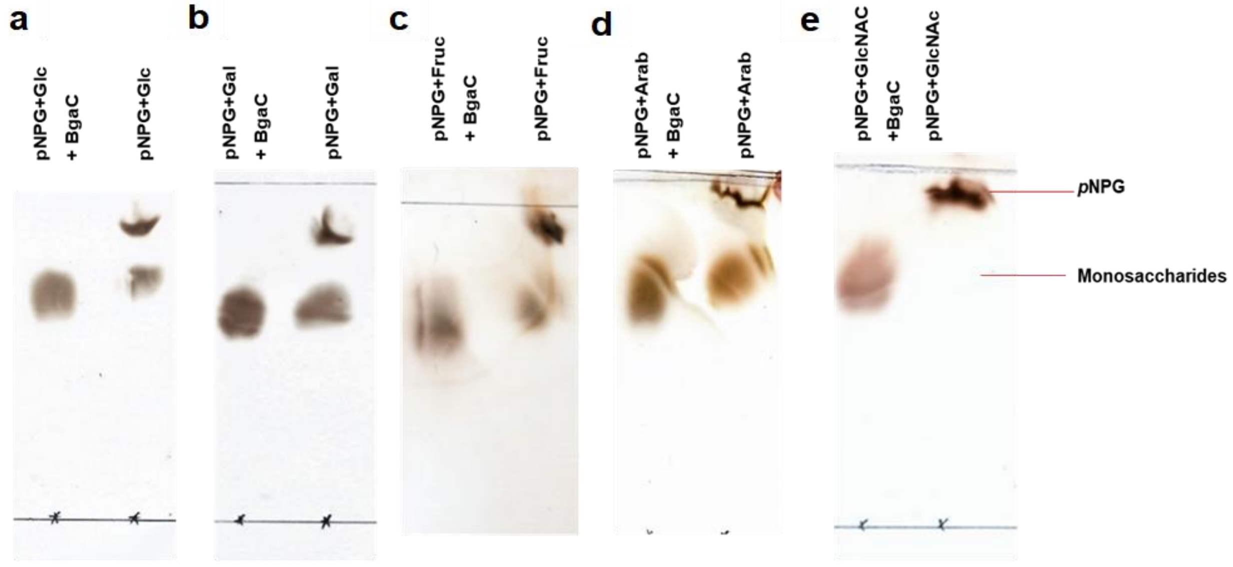
a
b
c
d
e
pNPG+Glc + BgaC
pNPG+Glc
pNPG+Gal + BgaC
pNPG+Gal
pNPG+Fruc + BgaC
pNPG+Fruc
pNPG+Arab + BgaC
pNPG+Arab
pNPG+GlcNAC +BgaC
pNPG+GlcNAc
pNPG
Monosaccharides

Fig. S5

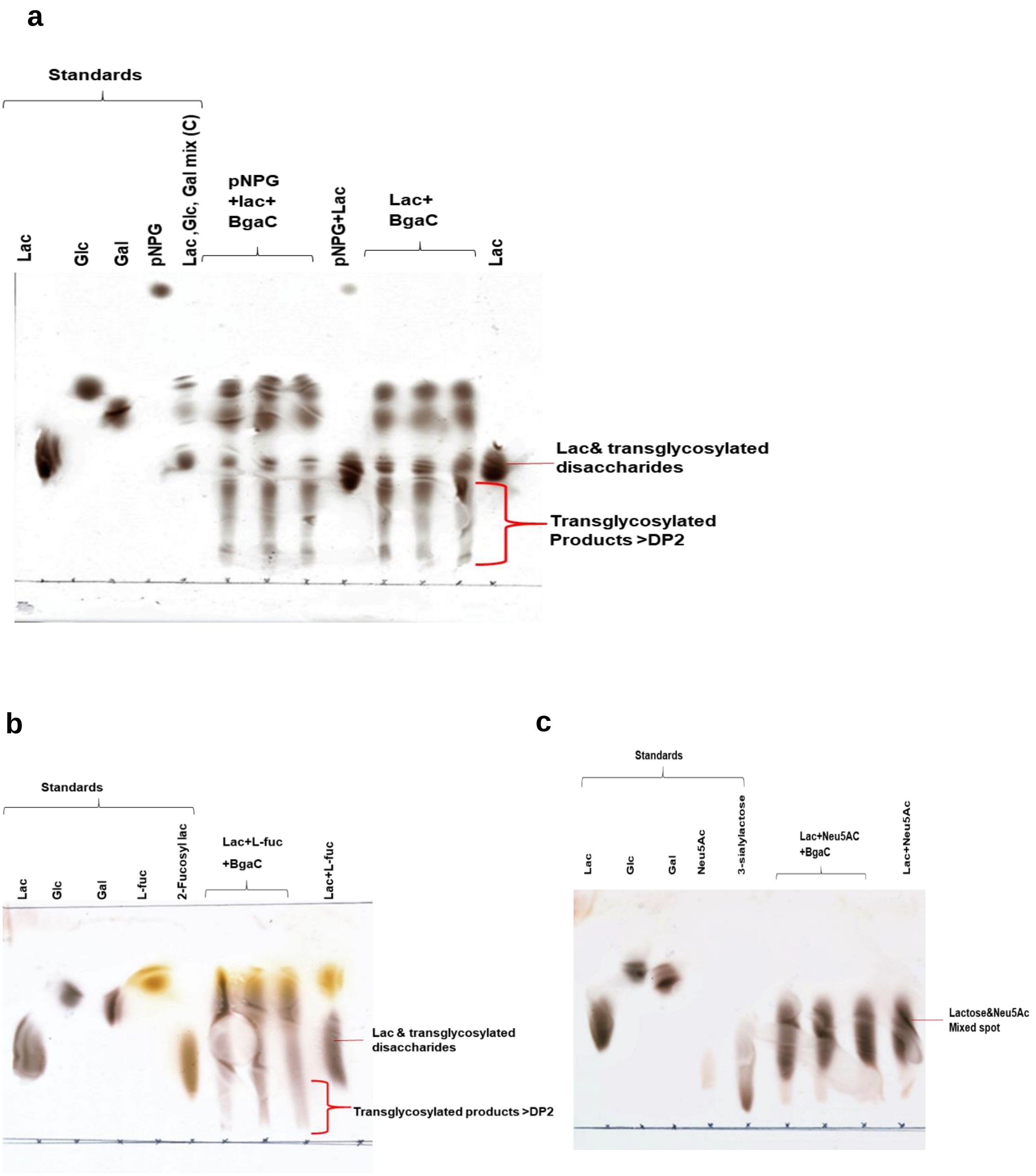
a
Standards
Lac
Glc
Gal
pNPG
Lac ,Glc, Gal mix (C)
pNPG
+lac+
BgaC
pNPG+Lac
Lac+
BgaC
Lac
Lac& transglycosylated
disaccharides
Transglycosylated
Products >DP2
b
Standards
Lac
Glc
Gal
L-fuc
2-Fucosyl lac
Lac+L-fuc
+BgaC
Lac+L-fuc
Lac & transglycosylated
disaccharides
Transglycosylated products >DP2
c
Standards
Lac
Glc
Gal
Neu5Ac
3-sialylactose
Lac+Neu5AC
+BgaC
Lac+Neu5Ac
Lactose&Neu5Ac
Mixed spot

Fig. S6

Table S1 Bacterial strains, Plasmid and Fosmids

**Bacterial strains, plasmids and fosmids**	**Genotype/ feature**	**Sources**
E. coli strains		
BL21(DE3)	*E. coli* str. B F^{-} *ompT gal dcm lon* $hsdS_B$ ($r_B^{-}m_B^{-}$) λ(DE3 [*lacI lacUV5-T7p07 ind1 sam7 nin5*]) [$malB^{+}$]$_{K-12}$($λ^{S}$)	Novagen
EPI300	F^{-} $λ^{-}$ *mcrA* Δ(*mrr-hsdRMS-mcrBC*) Φ80d*lacZΔM15* Δ*(lac)X74 recA1 endA1 araD139* Δ*(ara, leu)7697 galU galK rpsL* (Str^{R}) *nupG' trfA dhfr*	Epicentre
T7 express	*fhuA2 lacZ::T7 gene1 [lon] ompT gal sulA11 R(mcr- 73::miniTn10--* Tet^{S}*)2 [dcm] R(zgb-210::Tn10--* Tet^{S} *endA1* Δ*(mcrC-mrr)114::IS10*	NEB (New England Biolabs)
Plasmids and Fosmids		
pET101	T7 promoter expression vector, Amp^{R}	Invitrogen
pLysS	*T7p20* ori_{p15A}, Cm^{R}	Novagen
pCC1FOS	Fosmid, Cm^{R}	Epicentre
pDMg1a	pET101harbouring BAD_1582 with C-terminal His-tag, Amp^{R}	This study
